# FOXO transcription factors differ in their dynamics and intra/intermolecular interactions

**DOI:** 10.1016/j.crstbi.2022.04.001

**Published:** 2022-04-27

**Authors:** Emil Spreitzer, T. Reid Alderson, Benjamin Bourgeois, Loretta Eggenreich, Hermann Habacher, Greta Brahmersdorfer, Iva Pritišanac, Pedro A. Sánchez-Murcia, Tobias Madl

**Affiliations:** a Gottfried Schatz Research Center for Cell Signaling, Metabolism and Aging, Molecular Biology and Biochemistry, Medical University of Graz, Graz, Austria; b Division of Physiological Chemistry, Otto-Loewi Research Center, Medical University of Graz, Graz, Austria; c BioTechMed-Graz, Graz, Austria

**Keywords:** Transcription factors, Transcription activation domain, NMR spectroscopy, Protein dynamics, Spin relaxation, Molecular dynamics, TF, transcription factor, FOXO, Forkhead box O, SGK, serum and glucocorticoid-induced kinase, PP2A, protein phosphatase 2A, DBE, DAF-16 family member binding element, IRE, insulin response element, MD, molecular dynamics, IGF-1, insulin-like growth factor, NMR, nuclear magnetic resonance, NOE, nuclear Overhauser effect, RMSD, root-mean -square deviation, RMSF, root-mean-square-fluctuation, CSP, chemical shift perturbation, JNK, Jun N terminal kinase, TAD, transactivation domain

## Abstract

Transcription factors play key roles in orchestrating a plethora of cellular mechanisms and controlling cellular homeostasis. Transcription factors share distinct DNA binding domains, which allows to group them into protein families. Among them, the Forkhead box O (FOXO) family contains transcription factors crucial for cellular homeostasis, longevity and response to stress. The dysregulation of FOXO signaling is linked to drug resistance in cancer therapy or cellular senescence, however, selective drugs targeting FOXOs are limited, thus knowledge about structure and dynamics of FOXO proteins is essential. Here, we provide an extensive study of structure and dynamics of all FOXO family members. We identify residues accounting for different dynamic and structural features. Furthermore, we show that the auto-inhibition of FOXO proteins by their C-terminal trans-activation domain is conserved throughout the family and that these interactions are not only possible intra-, but also inter-molecularly. This indicates a model in which FOXO transcription factors would modulate their activities by interacting mutually.

## Highlights

• FOXO forkhead domains exhibit different dynamics.
• Helix 4 displays highest variations in dynamics among FOXO family members.
• The ability for forkhead - transactivation domain interaction is conserved among FOXOs.

## Graphical abstract

**Figure:**
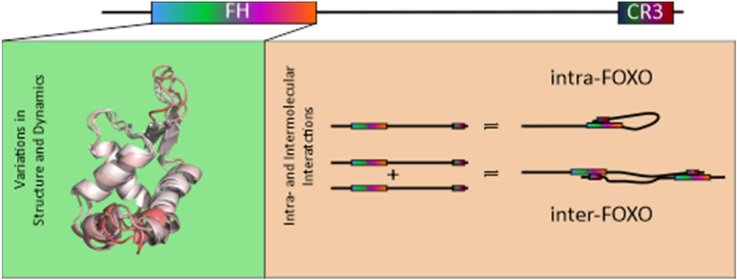

## 1 Introduction

Information encoded in the genome is interpreted by transcription factors (TF) to decide cell fates and establish complex body plans (Lambert et al., 2018; Spitz and Furlong, 2012). Besides functioning in development and differentiation, TFs control specific pathways in response to various stimuli such as immune response, stress response or nutrient sensing (Singh et al., 2014; Estruch, 2000; Hart, 2019). Typically, TFs recognize small 6–12 bp-long degenerate DNA sequences in regulatory regions, but the way that TFs impact transcription upon DNA binding varies considerably (Lambert et al., 2018; Wingender et al., 2015). While a few TFs act by directly recruiting RNA polymerase, most TFs are thought to contribute to transcription initiation by recruiting coactivators (Spitz and Furlong, 2012; Sikorski and Buratowski, 2009; Malik and Roeder, 2010; Juven-Gershon and Kadonaga, 2010; Taatjes, 2010). Mutations in TFs can contribute to cancer, autoimmunity, neurological disorders, developmental syndromes, diabetes, cardiovascular disease, and obesity, among others (Lee and Young, 2013). In cancer, TFs are overrepresented among oncogenes. When mutated, they unleash their oncogenic potential mainly by remaining in a permanently activated state (Furney et al., 2006; Pon and Marra, 2015), as for example TAL1 or c-Myc (Sanda et al., 2012; Littlewood et al., 2012; Nie et al., 2012; Lin et al., 2012). Loss-of-function mutations in TFs can act as tumor suppressors and are often the drivers of or are associated with various cancers (Sherr, 2004), with p53 being the most prominent (Olivier et al., 2002; Baker et al., 1990; Malkin et al., 1990).

Many TFs harbor small discrete domains, that allow them to bind to DNA (Harrison, 1991). Based on amino-acid sequence relationships and the three-dimensional structures of DNA-binding domains, TFs can be grouped into families (Harrison, 1991). TF families include helix-turn-helix (HTH) domain proteins (Brennan and Matthews, 1989), Zinc-finger domain proteins (Klug and Rhodes, 1987), the leucine-zipper coiled coil (Landschulz et al., 1988), or the winged helix, which is also known as Forkhead (FH) (Weigel and Jackle, 1990). Proteins harboring a FH domain are called FOX proteins (Kaestner et al., 2000). The FOX protein family comprises 19 subclasses from FOXA to FOXS with at least 50 members and fulfils an astonishing array of functions in development, physiology, cancer and cognition (Hannenhalli and Kaestner, 2009; Laissue, 2019; Katoh et al., 2013).

Given the large number of TFs that share the same folds and target DNA sequences, it is puzzling how homologs of TFs can fulfill biologically very different functions. Here, we focus on the FOXO TFs, which are considered to be tumor suppressors due to their functions in cell cycle arrest (Katayama et al., 2008; Medema et al., 2000; Tran et al., 2002; Dansen and Burgering, 2008), apoptosis (Brunet et al., 1999; Essafi et al., 2005), senescence (de Keizer et al., 2010), differentiation, DNA damage repair (Tran et al., 2002), and scavenging of reactive oxidative species (Jiramongkol and Lam, 2020; Essers et al., 2005; Eijkelenboom and Burgering, 2013; Burgering, 2008; van der Horst and Burgering, 2007; Gui and Burgering, 2021). The FOXO family consists of one gene in invertebrates, whereas there are four members in mammals, namely FOXO1, FOXO3, FOXO4 and FOXO6. FOXO activity is regulated by a wide range of external stimuli, such as insulin, insulin-like growth factor (IGF-1), other growth factors, neurotrophins, nutrients, cytokines and oxidative stress. These stimuli cause changes in post-translational modifications and subcellular localization of TFs, thereby regulating DNA-binding and transcriptional activity (Calnan and Brunet, 2008).

**Fig. 1:**
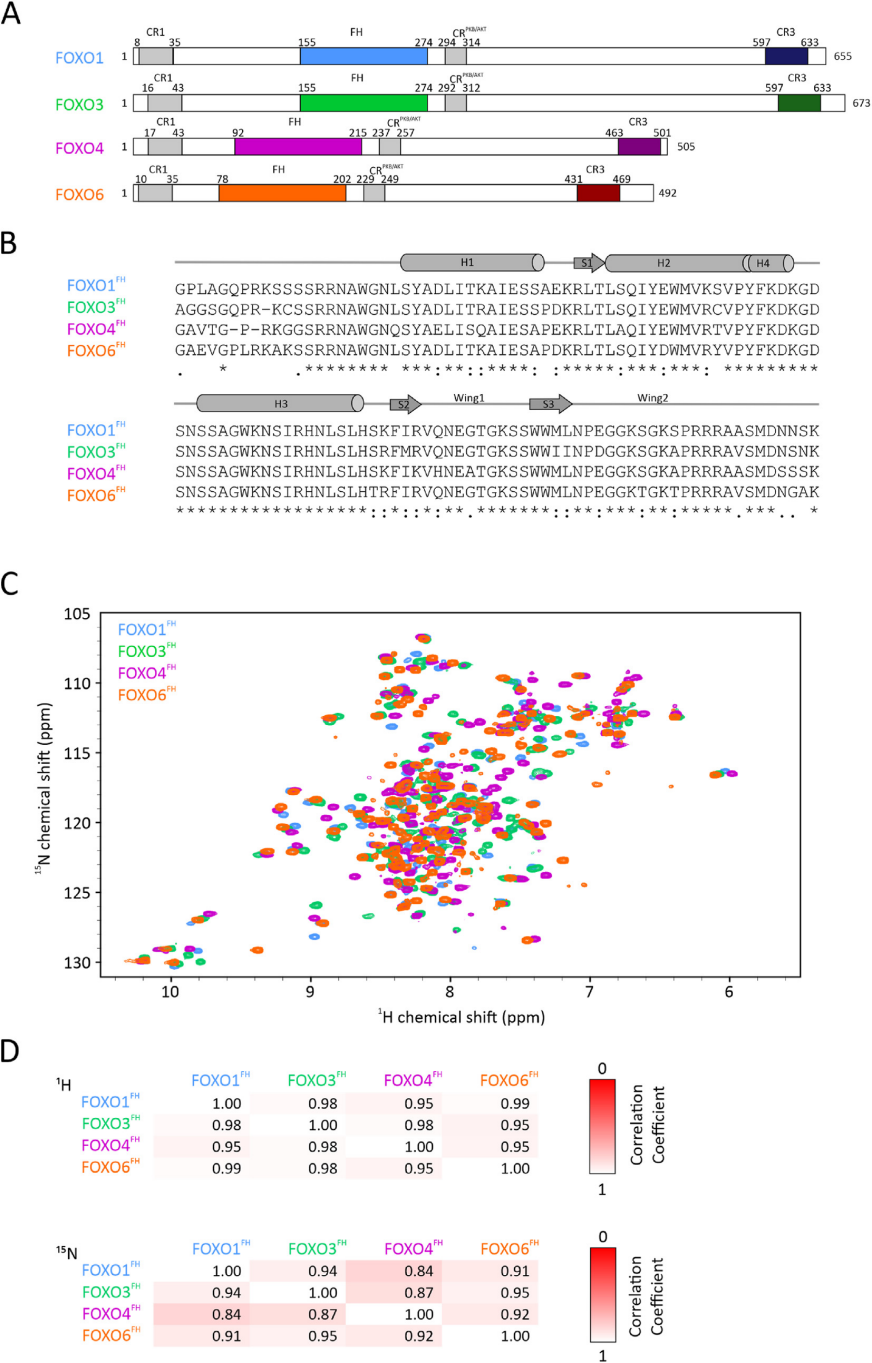
**Sequence and chemical shift comparison of FOXO family members.** (A) Domain architecture of human FOXO family members. (B) Sequence alignment of FOXO FH domains. Secondary structure elements are indicated above. (C) Overlay of 2D ^1^H,^15^N HSQC NMR spectra of ^15^N-labeled FOXO1^FH^, FOXO3^FH^, FOXO4^FH^ and FOXO6^FH^ at 300 ​μM colored in blue, green, magenta and orange, respectively. (D) Chemical shift correlation analysis of ^1^H chemical shifts (top panel) and ^15^N chemical shifts (lower panel) of FOXO FH domains. The colors reflect the correlation coefficient, ranging from 0 (red) to 1 (white).

All members of the FOXO family share a highly conserved FH domain, which is flanked by largely unstructured –N and –C terminal regions (Fig. 1A). Within these disordered termini, there are additional conserved regions (CR), termed CR1, CR^PKB/AKT^ (also denoted CR2) and CR3 (Obsil and Obsilova, 2008). CR1 and CR^PKB/AKT^ harbor phosphorylation sites for Akt/PKB that enhance 14-3-3 binding and subsequent nuclear exclusion, which represents one of the key regulatory mechanisms of FOXO activity (extensively studied and reviewed in (Calnan and Brunet, 2008; Brown and Webb, 2018; Almeida et al., 2007; Obsilova et al., 2005; Rinner et al., 2007)). FOXO6 differs from other members of the family as it is not subject to nucleo-cytoplasmatic shuttling (Calnan and Brunet, 2008; Jacobs et al., 2003). Recently, a mechanism for the regulation of FOXO transcriptional activity and specificity has been proposed, in which binding of the CR3 with the FH domain represses DNA binding. (Bourgeois et al., 2021; Kim et al., 2021).

Overall the sequences of FOXO proteins are more than 30% identical, with up to 90% identity within the DNA-binding FH domain (Fig. 1B) (Jacobs et al., 2003). The FH domain adopts a winged helix fold comprising four helices and three β-strands. Helix 1 (H1) is connected to H2 via a loop region and a short β-strand 1 (Fig. S1). H2 and H3 are connected by a region, that harbors a short helix (H4) and a five amino acid insertion, which is not present in any other FOX family. Two anti-parallel β-strands are C-terminal to the FH domain. H3 is responsible for DNA binding and the amino-acid sequence is fully conserved in all FOXO family members. Furthermore, all FOXO proteins recognize the same consensus DNA sequences 5′-GTAAA(T/C)AA-3′, known as the DAF-16 family member binding element (DBE) (Furuyama et al., 2000; Biggs et al., 1999, 2001) and 5′-(C/A) (A/C)AAA(C/T)AA-3′, known as the insulin response element (IRE) (Biggs et al., 1999; Kops et al., 1999). Several structures of the FOXO1, FOXO3, and FOXO4 FH domains in the free state and in presence of DNA have been solved in the last two decades by means of NMR spectroscopy and X-ray crystallography (Weigelt et al., 2000; Wang et al., 2008; Psenakova et al., 2019; Brent et al., 2008; Singh et al., 2017; Li et al., 2021a; Tsai et al., 2007; Boura et al., 2010). Overall, FOXO1, FOXO3 and FOXO4 share similar, yet slightly different structures. Whether these structural variations are due to the different conditions used in the studies (e.g. crystal vs. in-solution) is unclear. At present, there is no structural information for FOXO6.

FOXO function is often described as collective family function (Schmitt-Ney, 2020). This is exemplified by the study of O'Neill et al. in which the authors observed increased autophagy and muscle mass loss after the deletion of either the muscle insulin-receptor or IGF1-receptor, which is rescued by the combined loss of FOXO1, FOXO3 and FOXO4, but not if single FOXOs are deleted (Schmitt-Ney, 2020; O'Neill et al., 2016). Simultaneous deletion of FOXO1, FOXO3 and FOXO4 gave rise to tissue specific tumors, which is consistent with the tumor suppressive functions of FOXOs. Yet, a single- or double FOXO family knockout (KO) did not result in tumor formation, which indicates redundant functions among FOXO members (Paik et al., 2007).

Systematic KO studies in mice of FOXO isoforms revealed that FOXO1-KO is embryonal lethal, due to incomplete vascular development, whereas FOXO3- and FOXO4-KO mice were viable but grossly indistinguishable from their littermate controls. FOXO3-KO females additionally showed age-dependent infertility and had abnormal ovarian follicular development (Hosaka et al., 2004). The importance for FOXO1 for vascular development was confirmed later in depth (Wilhelm et al., 2016). KO studies of FOXO6 in mice revealed that FOXO6 activity is required for memory consolidation (Salih et al., 2012). FOXO4 can repress smooth muscle cells differentiation via repression of myocardin-mediated transcription, which is not recapitulated by FOXO1 and FOXO3 (van der Vos and Coffer, 2008; Nogueira et al., 2008).

Opposite effects in context of senescence have been observed for FOXO4 and FOXO1/3 (Bourgeois and Madl, 2018). In oncogene-induced senescence, the BRAF oncogene causes JNK mediated phosphorylation of FOXO4, which in turn leads to cell-cycle arrest and induction of senescence (de Keizer et al., 2010). In agreement with this, inactivation of FOXO4 in mice showed bypass of cellular senescence and oncogenic-BRAF induced melanoma formation (Dankort et al., 2009). Strikingly, opposite effects have been observed in cardiac microvascular endothelial cells, where i) FOXO3 overexpression suppressed senescence by increasing catalase and superoxide dismutase activities and ii) FOXO3 inactivation even accelerated senescence (Qi et al., 2015). In mouse embryonic fibroblasts it was shown that via Akt inactivation and consequent FOXO1 and FOXO3 activation, senescence was inhibited despite increased levels of ROS. Consistently, inactivation of either FOXO1 or FOXO3 restores premature senescence of Akt-deficient mouse embryonic fibroblasts (Nogueira et al., 2008).

At the atomic level the structure of the FOXO family members are highly similar when bound to DNA. However, the structures reveal different networks of hydrogen-bonds and water-mediated interactions in the major groove of the DNA (Obsil and Obsilova, 2011). FOXO4 adopts a different conformation than FOXO1 and FOXO3 upon DNA binding, in which Ser142 of FOXO4 interacts with the phosphate groups of the DNA backbone (Boura et al., 2010; Obsil and Obsilova, 2011). However, although differences in FOXO function could arise from differences in FOXO structures, the structural similarity among the FOXO FH domains suggests otherwise. Different FOXO functions could arise from the internal dynamics and allostery within FOXO FH domains, which may affect the binding to DNA and other interaction partners.

Here, we aimed to reveal whether variations in the structures and dynamics of the FOXO FH domains can be detected and if these differences affect key FOXO interactions. To this end, we studied the structures and dynamics of the FH domains of all human FOXO members (FOXO1, FOXO3, FOXO4, and FOXO6) in solution using NMR spectroscopy under identical experimental conditions, and extensive all-atom molecular dynamics (MD) simulations. We identified regions in FOXO FH domains that differ in terms of their dynamics, and we determined that these differences are due to slight variations in amino acid sequence. Furthermore, we show that the intra-molecular interactions between the FH and transactivation domains (TADs) recently discovered in FOXO3 and FOXO4 (Bourgeois et al., 2021; Kim et al., 2021; Wang et al., 2008) are also conserved in FOXO1 and FOXO6. Strikingly, we found that these interactions take place between FH domains and TADs of different FOXOs, provide the possibility of heterodimeric interaction with putative biological function. Among the FOXOs, FOXO3 shows interesting features as it is the member with the highest degree of flexibility within the FH domain and the weakest binding to its TAD. Taken together, our study provides essential clues for better understanding of functional difference of FOXO proteins in a variety of pathways.

## 2 Results

Since the sequence of all FH domains within the FOXO proteins is conserved to more than 90%, the structures are expected to be highly similar. Yet it was shown, that there are structural variations between FOXO1, FOXO3 and FOXO4 (Psenakova et al., 2019). Thus, we aimed to further elucidate structural similarities and differences of the FH domains of all FOXO family members. We first recombinantly expressed and purified all FH domains with ^15^N isotope labelling and recorded ^1^H, ^15^N heteronuclear single-quantum coherence (HSQC) NMR spectra (Fig. 1C). Given the high sequence identity amongst the four FOXO FH domains, one would expect that also their ^1^H,^15^N HSQC spectra are highly similar. Indeed, the correlation coefficients for both ^1^H and ^15^N chemical shifts (Fig. 1D and Fig. S1) showed high similarities between FOXO3^FH^ and FOXO6^FH^, whereas FOXO1^FH^ and FOXO4^FH^ are less similar, based on chemical shifts.

**Fig. 2:**
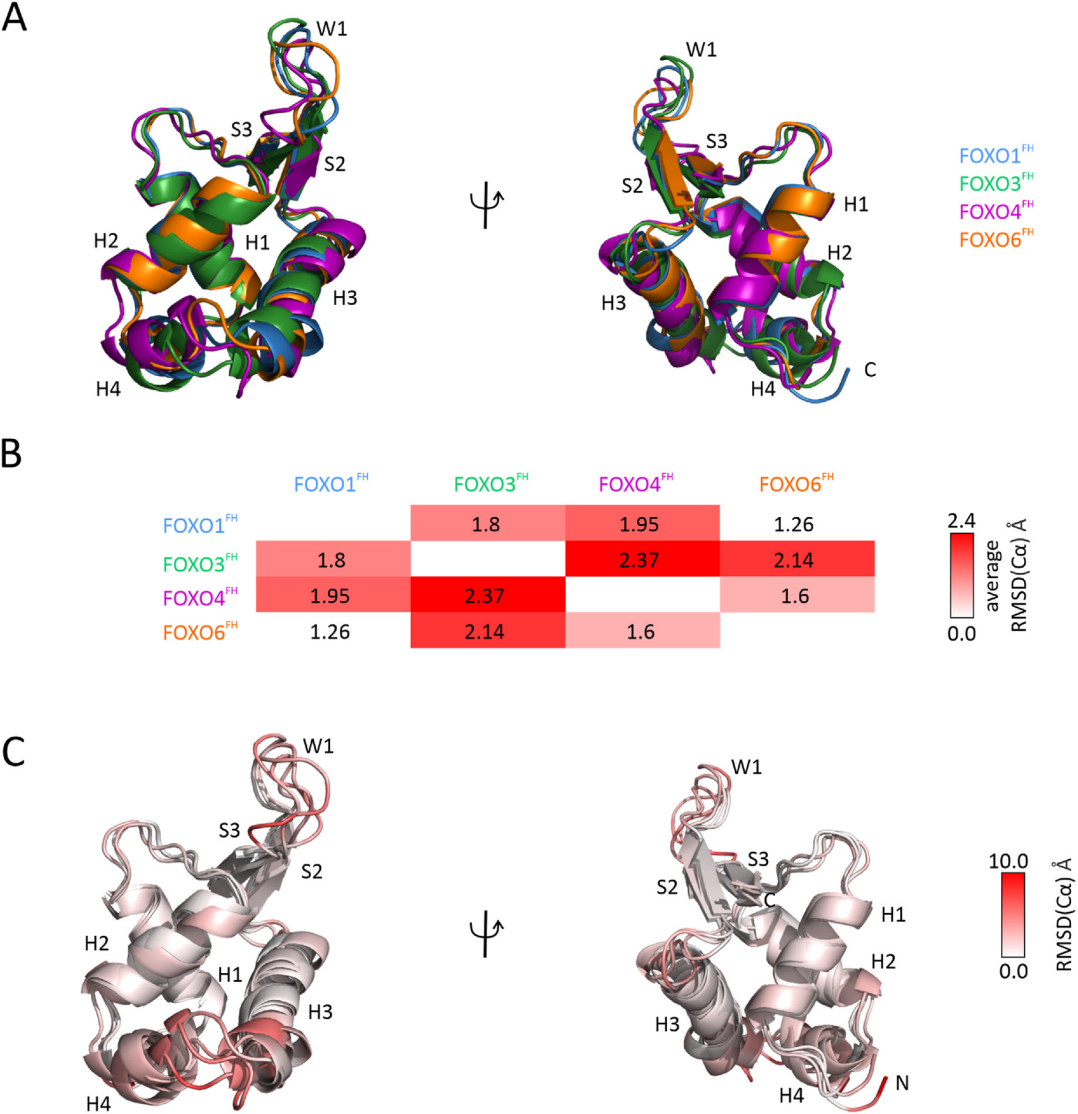
**FOXO6 structural models and FOXO structural comparison.** (A) Overlay of FH domains of FOXO1 (PDB ID: 6QVW), FOXO3 (2K86), FOXO4 (1E17) and FOXO6, colored in blue, green, magenta and orange respectively. (B) Overall average pair-wise backbone RMSD between FOXO proteins from 0 ​Å (white) to 2.4 ​Å (red). (C) The same structures in (A) colored given by their Cα RMSD (from 0 ​Å in white to 10 ​Å in red).

To compare the static structures of all FOXOs, we first predicted the, yet unsolved, FOXO6^FH^ structure using the machine learning-aided structure prediction tool AlphaFold2 (Fig. S2A and Fig. S2B). Like the other FOXO proteins, the FOXO6 FH domain shows the winged-helix fold (Fig. 2A). Since the previously reported solution structures of the FOXO1/3/4^FH^ domains (PDB ids: 6QVW (Psenakova et al., 2019), 2K86 (Wang et al., 2008) and 1E17 (Weigelt et al., 2000), respectively) were solved with different construct lengths, we focused here on the comparison of residues present in all structural models (sequence alignment in Fig. 1A). Additionally, the experimental structural models were determined using different NMR refinement methods. Thus, and to allow a direct comparison, we first ran all-atom MD simulations on the μs-scale to obtain force-field equilibrated models for all FH domains. As outcome, we observed that the force-field equilibrated models of FOXO1^FH^ and FOXO6^FH^ are the most similar (Fig. 2B, root-mean-square deviation (RMSD) of 1.3 ​Å). This is in agreement with the chemical shift analysis in which the FH domains of FOXO1, FOXO3 and FOXO6 display the most similar chemical shifts (Fig. 1D). FOXO3^FH^ shows the highest differences with the FH domains of both FOXO4 (2.4 ​Å) and FOXO6 (2.2 ​Å) (Fig. 2B). The dissimilarity of FOXO3^FH^ and FOXO4^FH^ is also observable in the chemical shift analysis (Fig. 1D). Next, we analyzed FH regions with differential structural variability amongst the four FH domains along the MD simulation (Fig. 2C and Fig. S2). In Fig. 2C we show a superimposition of all FOXO FH domains with the secondary structure color coded according to the backbone RMSD. Most structural variations are located in the region connecting H2 and H3, including helix H4, on the N-terminus of H3. Further differences are observable in the loop connecting the β-strands S2 and S3 (Fig. 2C).

In summary, we found, as expected, that the FOXO6^FH^ adopts a similar fold as all other FOXO family members. However, despite the high sequence identity, our MD simulations studies show that there are conformational differences within the FH domains of the FOXO proteins. The most variations are found in H4, the transition to H3 and in the loop regions connecting the β-strands S2 and S3.

### 2.1 NMR relaxation studies of forkhead domains reveal differences in FOXO dynamics

**Fig. 3:**
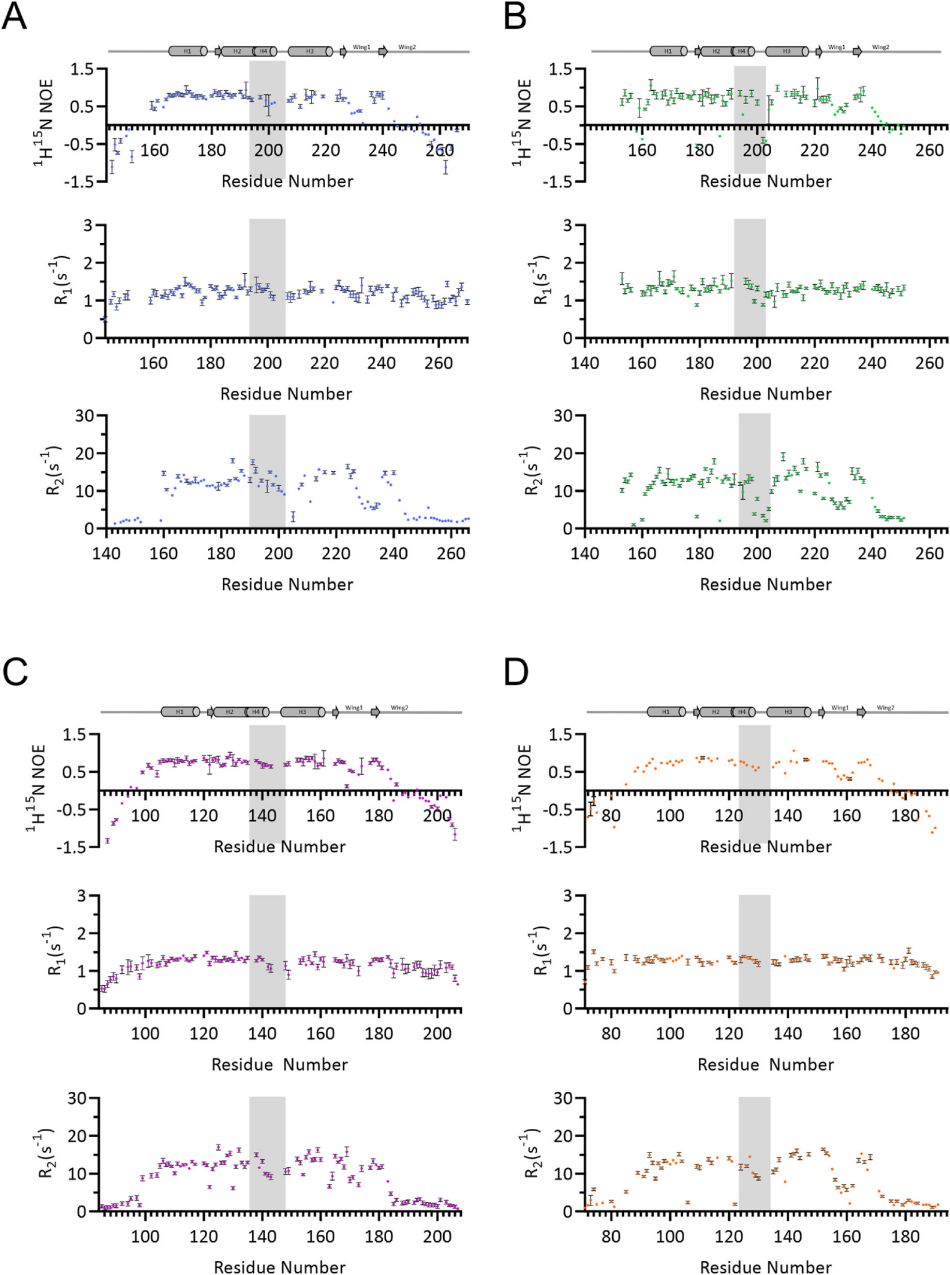
**Backbone dynamics of FOXO FH domains.** Backbone amide NMR spin relaxation data for A) FOXO1^FH^, B) FOXO3^FH^, C) FOXO4^FH^, D) FOXO6^FH^ each with ^15^N{^1^H} heteronuclear NOE in the top panel, ^15^N *R*_1_ in the middle panel and ^15^N *R*_2_ relaxation rates in the lower panel measured at 298 ​K at a static magnetic field strength of 600 ​MHz. *R*_*2*_ rates were converted from *R*_1ρ_ using equation (3).

A comparison of structural snapshots can give insights into static differences. In order to obtain a more in-depth comparison of the backbone dynamics of the FH domains, we measured ^15^N longitudinal (*R*_*1*_) and transverse (*R*_*2*_) relaxation rates and the steady-state ^15^N,^1^H-Nuclear Overhauser Effect (NOE) for all FOXO FH domains. Heteronuclear ^15^N{^1^H} NOE values are indicators of the motions of amide bond vectors on the ps/ns timescale: values close to the theoretical limit of 0.83 ​at a static magnetic field strength of 600 ​MHz suggest rigidity whereas lower values indicate increased local flexibility. The regions that adopt stable secondary structure elements show ^15^N{^1^H} heteronuclear NOE values above 0.5 for all FOXO FH domains (lower flexibility), whereas the terminal ends show negative ^15^N{^1^H} heteronuclear NOEs, indicating high flexibility with rapid ps/ns motions (Fig. 3A–D upper panels). In general, residues located on the wing 1 (W1) and region connecting H2 and H3, which contains H4, exhibit lower ^15^N{^1^H} heteronuclear NOEs compared to the other folded regions. Interestingly, in case of FOXO3^FH^, several residues such as Asn201 and Ser202 in H4 show negative ^15^N{^1^H} heteronuclear NOEs. Furthermore, Leu178, located in the loop connecting H1 and H2, and Trp187, in H2, show negative ^15^N{^1^H} heteronuclear NOE values, indicating high flexibility.

All FOXO FH domains show similar ^15^N *R*_1_ rates indicating that the sub-ns backbone dynamics are comparable (Fig. 3A–D, middle panels). In line with the ^15^N{^1^H} heteronuclear NOE analysis, the ^15^N *R*_2_ rates are decreased for residues within H4 of all FOXO FH domains as compared to the surrounding structured regions, suggesting that H4 is not as stably formed and that the N–H bond vectors in this region are relatively dynamic on the ps timescale. Notably, the H4 of FOXO1 appears to be the most rigid when compared to the other FOXO family members. Interestingly, the Trp residue in H2 exhibits different dynamics in the FOXO isoforms. In contrast to FOXO1 and FOXO6, the Trp residue in H2 shows low ^15^N *R*_2_ rates in FOXO3 and FOXO4. In addition, the Leu residue located in the loop connecting H1 and H2 shows lower *R*_2_ rates in FOXO3 and FOXO4, indicating that this residue within the loop is highly flexible. Finally, we note that W1 and W2 in all FOXO isoforms have low *R*_2_ rates and ^15^N{^1^H} heteronuclear NOE values that indicate rapid ps-ns motions. However, in the case of FOXO4, W1 has relatively elevated *R*_2_ rates that may indicate that this region is either more rigid than the other isoforms or may exhibit microsecond timescale motions that were not effectively suppressed by the spin lock applied during the experiment.

In summary, the relaxation rates and steady-state ^15^N{^1^H} heteronuclear NOE overall reflect the known secondary structure elements and flexible regions. Interestingly, helix H4, the H2–H3 loop and W1 are the most flexible regions within FOXO FH domains. Especially the H4 of FOXO3 appears to be highly dynamic.

### 2.2 Molecular dynamics simulations reveal variable dynamics in conserved regions

Our NMR relaxation measurements outlined similarities and differences in the backbone dynamics of FH domain. To obtain detailed insight into the molecular details of the underlying motional processes, we performed extensive all-atom MD simulations of each of the FH isoforms in solution in which (three independent 0.5 μs simulations were conducted per system for a total of 1.5 μs simulation time). As observed with our prior NMR-based analysis, most of the secondary structure elements including H1, H2, H3, as well as S1, S2 and S3 remain stable in all isoforms (Figs. S4A and S4B). During the simulation, however, H4 only adopts an α-helical conformation 45% of the time. For the rest of the simulation, H4 switches from an α-helix to a 3–10 helix or loses any secondary structure, which is in agreement with our NMR dynamics data that point to more rapid dynamics in H4 than expected for a stable, fully formed helix.

**Fig. 4:**
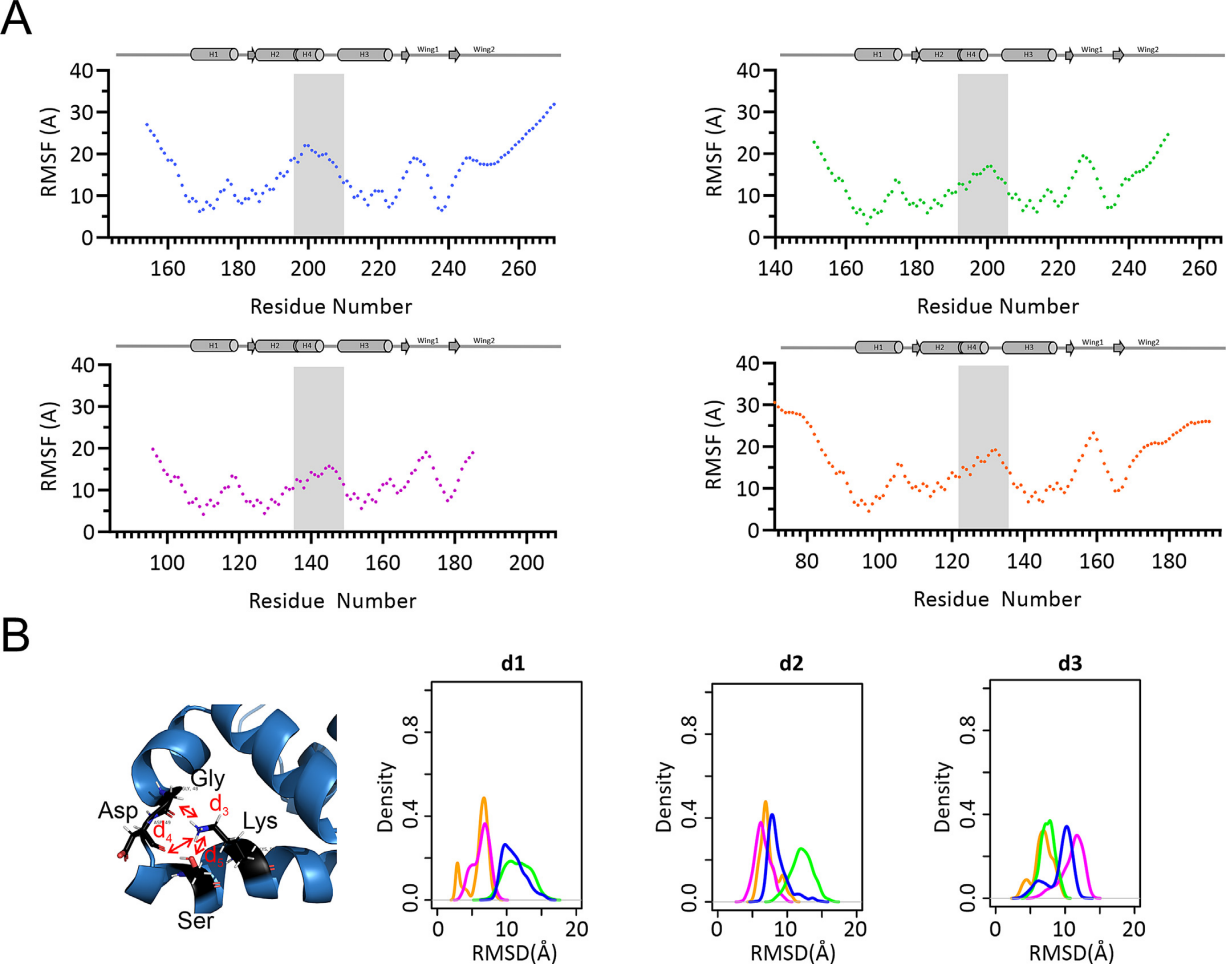
**Molecular dynamics simulations of FOXO FH domains.** (A) Root mean square fluctuation of FH domains of FOXO1 in blue, FOXO3 in green, FOXO4 in magenta and FOXO6 in orange over 1500ns of all-atom MD simulation. (B) Distances between H4 and H3 measured throughout the simulation of FOXO1 (blue), FOXO3 (green), FOXO4 (magenta) and FOXO6 (orange) respectively over 1500ns MD simulation. The distances d_1_, d_2_ and d_3_ are defined in the structure of FOXO1 shown above and applied to the corresponding residues in FOXO3, FOXO4 and FOXO6.

To follow the molecular motions on a residue-specific level, we calculated the root-mean-square-fluctuation (RMSF) for each of the FH isoforms (Fig. 4A). As expected, based on our NMR data, the highest RMSF values were observable in the N- and C-termini, in the H1–H2 loop, in W1, and in H4 for each FOXO-FH domain.

In order to characterize the structural variability within the FOXO family we examined in more detail the region between H4 and the N-terminus of H3, which displayed variability in the static structures and NMR relaxation measurements. Therefore, we monitored three distances throughout the MD simulations between the nitrogen of the side chain in Lys210 (NZ) and (i) the carboxyl group of Gly201 (CO) (termed d_1_), (ii) the carboxyl group of Asp202 (CO) (d_2_), and (iii) the hydroxyl group of Ser206 (OH) (d_3_). These FOXO1 residues correspond to Gly198, Asp199, Ser203 and Lys207 in FOXO3, to Gly142, Asp143, Ser147 and Lys151 in FOXO4, and to Gly129, Asp130, Ser134 and Lys138 in FOXO6, respectively (Fig. 4B). In agreement with the low R_2_ rates and low ^15^N{^1^H} heteronuclear NOE values in FOXO3, the distance distribution of d_1_ and d_2_ is broader in FOXO3 compared to other FOXOs. The distance between the carboxyl group of Asp199 and the nitrogen of the side chain in Lys207 is significantly larger in FOXO3.

The high structural stability shown by the first helical turn in H4 in all isoforms (Tyr196-Lys200 in FOXO1^FH^; Tyr193-Lys197 in FOXO3^FH^; Tyr137-Lys141 in FOXO4^FH^; and Tyr124-Lys128 in FOXO6^FH^) can be explained by the formation of a stable hydrogen bond between the phenyl hydroxyl group (OH) of Tyr196 and the amino group of Lys200 (NZ) (distance d_4_, Fig. S4F) as well as a salt bridge between the carboxyl group of Asp199 (OD1) and the amino group of Lys200 (NZ) (distance d_5_).

Additionally, we measured the relative orientation of H4 towards H2 along the MD simulations (Fig. S4F) by measuring the distance between the amino group of Lys to the carboxyl group of the C-terminal residue of H2 (d_6_). We found out that in FOXO6 the amino group of Lys is closer to H2 compared to the other FOXO isoforms, which behave similarly throughout the MD simulations.

Thus, H4 in FOXO6 displays the most rigidity during our MD simulations based on the smallest values for the examined distance distributions, as well stable secondary structure (Fig. S4), as compared to the other FOXO FH domains. The NMR spin relaxation data for FOXO6 are consistent with a relatively stable helix, with {^15^N}^1^H heteronuclear NOE values above 0.7 and *R*_2_ rates above 10 s^−1^ (Fig. 3). By contrast, based on our NMR data and MD simulations, H4 in FOXO3 appears to be more flexible than any of the other H4 regions in FOXO FH domains.

In summary, our MD simulations allowed us to identify interactions at the atomic level and to enlighten the molecular details of variations observed in our NMR dynamics studies.

### 2.3 FOXO FH domains present several non-conserved repeated W-[2-5]-[S/T/C/Y] motifs

**Fig. 5:**
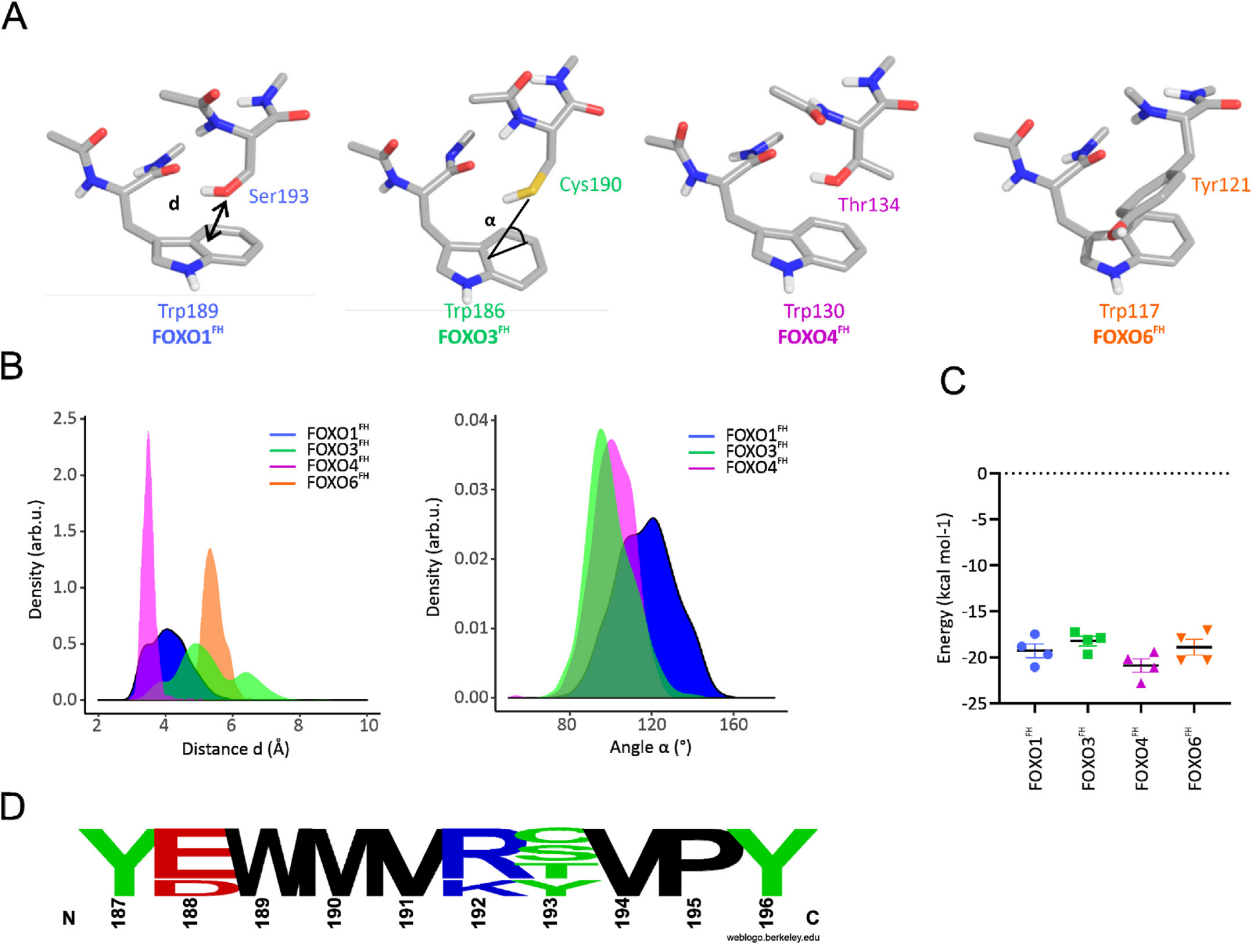
**Interaction between variable residue and conserved Trp occur at different strengths.** (A) Representative structure of the W-[2-5]-[S/T/C/Y] motif in the four isoforms and definition of the distance d (indicated in FOXO1) and the angle α. (indicated in FOXO3 (B) Distribution of the distance d (Å) and the angle α (degree) along the MD simulations (1.5 µs/system). (C) Interaction energy (kcal mol^−1^) between the two residues Xaa and W in each of the isoforms. Five geometries were taken for the calculation of the interaction energy at the level of theory APFD/6-311+G(2d,p). (D) Sequence of W-. (Spitz and Furlong, 2012; Singh et al., 2014; Estruch, 2000; Hart, 2019)-[S/T/C/Y] motif in FOXO isoforms.

Given the variations between the FOXO isoforms in regions that are conserved on a sequence level found in the static snapshots, NMR-based dynamics measurements and atomistic MD simulations, we sought to understand which sequence variation is responsible for such differences. Thus, we mapped the non-conserved residues within the FH domain onto the structure of each FOXO FH isoform. Most of the non-conserved residues are located in regions that are highly accessible to the solvent in each of the FOXO FH isoforms. Thus these non-conserved residues may not be the cause for intramolecular structural and dynamical changes as those found in H4 in our NMR studies. While searching for non-conserved residues, we identified several 3D-motifs in which the side chain of a tryptophan interacts with the side chain of Ser (FOXO1), Thr (FOXO4), Cys (FOXO3) or Tyr (FOXO6) (Fig. 5A). In all cases, the two interacting residues are separated by 2–5 residues W-[2-5]-[S/T/C/Y].

In order to obtain insight into the structural stability of the Xaa-Trp interaction in the motifs Xaa-(Yaa)_3-5_-W, we monitored the distance during the MD simulations between the heteroatom of the side chain of the terminal residue Xaa (Ser/Thr/Cys) and the center of the indole ring of the interacting Trp (Fig. 5A and B). As observed in Fig. 5B, the alcohol oxygen of Ser/Thr is closer to the aromatic system of Trp than the sulfur of Cys. The furthest distance was found between Trp and the phenol oxygen of Tyr in FOXO6^FH^, although we note that the aromatic ring of Tyr was typically situated near the Trp residue in an edge-to-face conformation. Finally, the distributions of the angle α (see Fig. 5A) in FOXO1 and FOXO3 are larger than FOXO4, indicating that the Xaa-Trp interaction in FOXO4 is more stable, likely due to the additional methyl group of the Thr residue.

In addition, we also computed the interaction energy between the former two side chains (Fig. 5C), using quantum mechanics. Briefly, the interaction energy was calculated following the counterpoise method, which includes a basis set superimposition error correction (Jensen, 2010). As shown in Fig. 5C, Cys is the residue with the smallest interaction energy (−16.00 ​± ​1.17 ​kcal ​mol^−1^), whereas the other energies are quite similar. The interaction of Tyr with Trp has a medium value and is not a face-to-face π-π stacking interaction but an edge-to-face interaction. Thus, our MD simulations and quantum mechanical calculations have shown variable interaction strengths between the Xaa-Trp residues within the W-[2-5]-[S/T/C/Y] motifs of FOXO FH domains, with FOXO4 having the most favorable interaction (Thr-Trp).

Looking at the full-length protein sequences, the W-[2-5]-[S/T/C/Y]motif is repeated across the sequence of FOXOs, although to a varying degree between the four isoforms (Fig. S5A). Whereas in FOXO1 there are three Ser-Trp motifs, in FOXO3 there is only one Cys-Trp; in FOXO4 there are two Thr-Trp motifs, and in FOXO6 two of Tyr-Trp. Interestingly, all isoforms have multiple conserved Ser-Trp motifs within the FH domain (at positions Trp160 and Ser164, Ser205/206 and Trp209 and Ser 212, Ser234 and Trp236 (FOXO1 numbering)). Notably their structural arrangement is not similar in all instances. Given the large number of Trp residues in the FH domain of all FOXOs we checked for evolutionary conservation of these residues. Indeed, the Trp residues within the FH domain are conserved in some tested model organisms. Interestingly, the variable residue within the W-[2-5]-[S/T/C/Y] motif is an Asn in *D. rerio* and in *C. elegans* (Fig. S5B). We cannot assign yet the impact of the repetition of such Xaa-(Yaa)_3-5_-W motifs and how the different residues Xaa might affect the interaction with Trp. We know that these motifs might not be related to the direct interaction with DNA as found in the available structures (PDB id. 3CO6, 2UZK, 3L2C). Interestingly, we found a population in our MD simulations of FOXO3^FH^ (and not in the other isoforms) where the N-terminal Trp157 interacts with Cys190 thereby displacing Trp186 which then interacts with Arg189. This suggests that there may be plasticity between the Xaa-Trp interactions within the repeated W-[2-5]-[S/T/C/Y] motifs in FOXO FH domains, and further investigations on the Xaa-Trp motifs are planned.

### 2.4 Back-folding of the CR3 on the FH domain is conserved in all FOXO family members

**Fig. 6:**
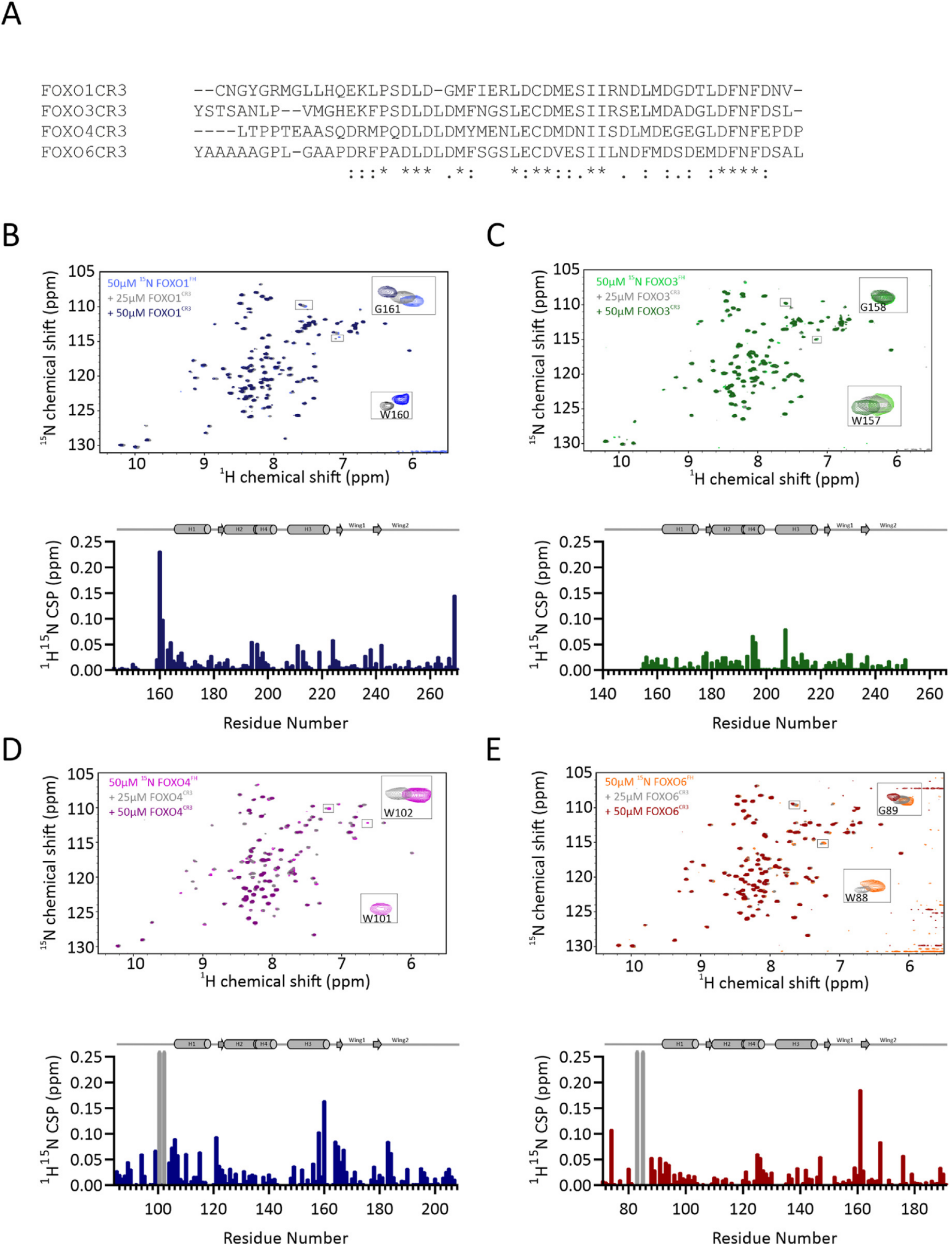
**NMR binding studies of intramolecular FH – CR3 interactions.** (A) Sequence alignment of the FOXO CR3s. (B–E) 2D ^1^H–^15^N HSQC spectra of 50 ​μM ^15^N-labeled FOXO1^FH^, FOXO3^FH^, FOXO4^FH^ and FOXO6^FH^ in absence (in blue, green, magenta, orange respectively) and presence of 25 ​μM (grey) or 50 ​μM of their corresponding CR3 (in dark blue, dark green, dark violet, dark red respectively). The insets show example cross-peaks from FH domain residues that are affected by CR3 binding. The lower panels show the quantification of ^1^H,^15^N chemical shift perturbations for FH domain residues in the presence of 50 ​μM CR3. Grey bars correspond to residues that are broadened upon binding. Missing values correspond to prolines or unassigned residues.

Transcription is a highly important cellular process and thus, DNA binding of transcription factors is tightly controlled. It has been shown for a few TFs, including p53, CCAAT/Enhancer-binding Protein β, FOXO3 and FOXO4, that their disordered regions can interact with their folded DNA-binding domains and regulate DNA binding (Bourgeois et al., 2021; Kim et al., 2021; Wang et al., 2008; Tafvizi et al., 2011; Krois et al., 2018; Lee et al., 2010). In the case of FOXO3 and FOXO4, the disordered CR3s bind to the FH domain on a surface overlapping with the DNA binding site (Bourgeois et al., 2021; Kim et al., 2021; Wang et al., 2008). This FH-CR3 binding interaction was shown to inhibit DNA binding in FOXO4 (Bourgeois et al., 2021). As both the FH domain and the CR3 are conserved in FOXO1 and FOXO6 (Fig. 6A), we hypothesized that this mechanism is also conserved in these proteins.

To test for the conservation of CR3 binding to the FH domain in other FOXO proteins, we titrated ^15^N isotope-labeled FOXO FH domains with their recombinantly expressed CR3. The addition of FOXO1^CR3^ to ^15^N labeled FOXO1^FH^ resulted in progressive chemical shift perturbations (CSPs), that are indicative of binding (Fig. 6B). Quantification of the CSPs showed that mainly residues N-terminal of H1 in FOXO1^FH^, including Trp160 and Gly161 are affected by FOXO1^CR3^ binding. Using a similar setup, we titrated FOXO6^CR3^ to ^15^N labeled FOXO6^FH^ and observed CSPs and line broadening of ^1^H,^15^N cross peaks of FOXO6^FH^ (Fig. 6E). Calculation of the CSPs shows that mainly the N-terminal Trp-Gly motif and H3 are affected by binding (Fig. 6E). Furthermore, we confirmed previously published results of the intramolecular interactions present in FOXO3 and FOXO4 between the FH domain and the CR3 respectively (Fig. 6C and D)- Thus, the CR3s in all FOXO proteins bind to their respective FH domains in solution when added in *trans*.

The CR3 of FOXO4 forms two transient α-helices upon binding to the FOXO4 FH domain and bends around H3 in a manner similar to DNA (Fig. S6A) (Weigel and Jackle, 1990; Kaestner et al., 2000; Hannenhalli and Kaestner, 2009). To identify the binding surface of the CR3s on their respective FH domains we plotted the CSPs on the structural models of the respective FOXO FH. The surfaces affected by CR3 binding include N-terminal parts of H1, H3 and H4 and is roughly similar in all FOXOs. However, we note that the CR3s from the different FOXO proteins bind with varying affinities to their respective FH domains (*vide infra*), and thus the smallest CSPs are observed in FOXO3, suggesting the weakest interaction.

Given that the CR3 from FOXO4 binds to the FH domain in an α-helical conformation, we hypothesized that the FOXO CR3s might already populate similar helical conformations in the free state. Since there are no high-resolution structures for the unbound CR3 regions, we used AlphaFold2 (Jumper et al., 2021) to predict the conformations of the CR3s based on their amino acid sequences. Despite slight differences especially in the terminal regions, the core part, which was shown for FOXO4^CR3^ to mediate FOXO4^FH^ binding, is highly similar in all FOXOs (Figs. S6A–D), although there are slight deviations in the terminal regions. To experimentally determine the propensity to form secondary structure elements we measured the ^15^N{^1^H} heteronuclear NOE and calculated the ^13^Cα secondary chemical shifts of the FOXO CR3 domains (Figs. S6A–D, S6A-D left and right panel respectively). In FOXO1 and FOXO3, there is a slight tendency of the CR3 to form α-helices in the same regions predicted by AlphaFold2, whereas FOXO4^CR3^ and FOXO6^CR3^ are largely devoid of residual secondary structure elements. These results indicate that while the intramolecular interaction of the FH domain with the CR3 is conserved throughout the whole FOXO family, the residual helical structure within the unbound CR3 is observed only in FOXO1 and FOXO3. The FH domains harbor a conserved N-terminal Trp-Gly motif that is especially affected in FOXO1, FOXO4 and FOXO6 upon binding to the CR3. These residues have been previously shown to be involved in intramolecular interactions in FOXO3 and FOXO4 (Bourgeois et al., 2021; Wang et al., 2008). Interestingly, in FOXO3 binding of the CR3 to the FH domain cause only weak chemical shift perturbations in this region, indicating a weaker binding. By contrast, FOXO1^CR3^ induced the largest CSPs upon binding to its FH domain and FOXO4^CR3^ and FOXO6^CR3^ caused line broadening in ^1^H,^15^N cross peaks of their respective FH domain, indicating stronger binding. Thus, while both FOXO1^CR3^ and FOXO3^CR3^ have residual helical structure in the unbound state, the binding affinity for their respective FH domains appears to be encoded both in the sequence of the CR3 and in the N-terminal Trp-Gly motif of the FH domain.

### 2.5 FH and CR3 of different FOXOs interact intermolecularly

The interface on the FOXO FH domains with respect to intramolecular CR3-binding is highly similar and conserved between FOXOs. Thus, we aimed to investigate possible intermolecular interactions between FOXO members. To address this, we used a similar approach as above and titrated ^15^N labeled FOXO FH domains with unlabeled CR3, in all possible combinations.

**Fig. 7:**
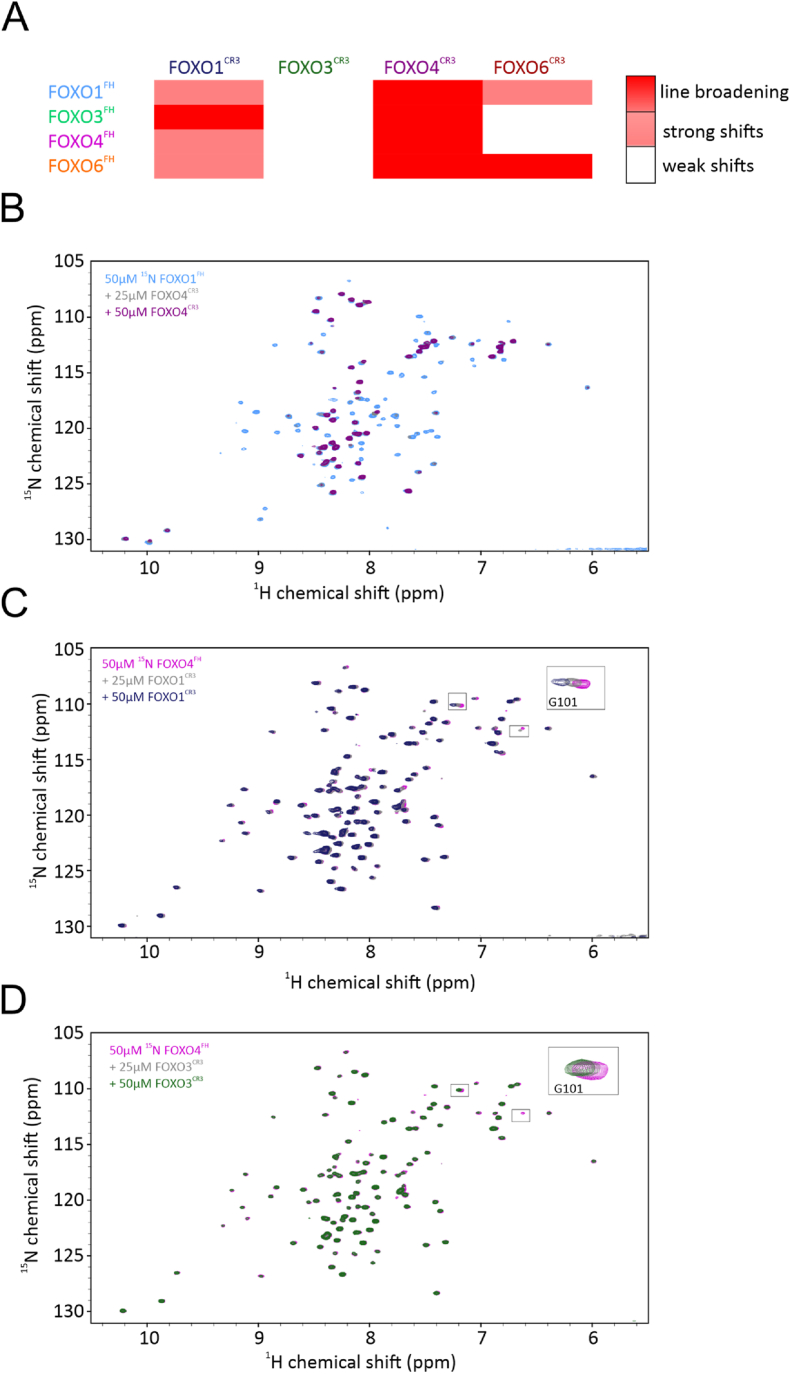
**Overview of NMR binding studies of intermolecular FH – CR3 interactions from different FOXO proteins.** (A) Overview of chemical shift titrations, categorized from weak chemical shift perturbations (white), and strong chemical shift perturbations (pink) to line broadening (red). B-D representative examples for interactions in weak chemical shift perturbations (B), strong chemical shift perturbations (C) and line broadening (D).

The addition of unlabeled FOXO^CR3^ to a solution of ^15^N labeled FOXO^FH^ resulted either in i) line broadening ii) strong CSPs or iii) weak CSPs of ^1^H,^15^N cross-peaks of the FH domain. Fig. 7 shows a summary of the results of the titration studies and representative examples for line broadening, strong- and weak CSPs. Line broadening was observed for ^1^H,^15^N cross-peaks of FOXO1^FH^, FOXO3^FH^ and FOXO6^FH^ in the presence of FOXO4^CR3^ and for FOXO3^FH^ in the presence of FOXO1^CR3^. Strong CSPs were observed for ^1^H,^15^N cross-peaks of FOXO1^FH^ in the presence of FOXO6^CR3^, of FOXO4^FH^ and FOXO6^FH^ in the presence of FOXO1^CR3^. Weak CSPs were observed ^1^H,^15^N cross-peaks of FOXO1^FH^, FOXO4^FH^ and FOXO6^FH^ in the presence of FOXO3^CR3^ and of FOXO3^FH^ and FOXO4^FH^ in the presence of FOXO6^CR3^ (Fig. S7).

Taken together, we show that intermolecular FH-CR3 interactions of different affinities can be detected between FOXO proteins. The strongest interaction is observed between the FOXO4^CR3^ and the FH domain of all FOXO proteins. In case of FOXO3^FH^ interestingly, the CR3 of FOXO1 and FOXO4 bind stronger as compared to its own CR3 and the CR3 from FOXO6. The FOXO3^CR3^ only causes minor chemical shift perturbations of ^1^H,^15^N cross-peaks of any FOXO FH domain. Overall, these results suggest that hetero-oligomeric FOXO complexes could possibly form via the binding of a CR3 domain from one FOXO isoform to the FH domain of another FOXO isoform. For instance, in the case of FOXO3, the weak intramolecular CR3-FH interaction may be outcompeted by an intermolecular CR3-FH interaction from FOXO1 and FOXO4.

## 3 Discussion

TFs are key for deciding cellular fates, responses to various stimuli and maintaining cellular homeostasis (Lambert et al., 2018). Commonly, TFs recognize small 6–12 bp-long degenerate DNA sequences and act either via directly recruiting the RNA polymerase or via recruitment of coactivators (Spitz and Furlong, 2012; Sikorski and Buratowski, 2009; Taatjes, 2010). Based on the sequences and three-dimensional structures of TFs, they can be grouped into distinct TF families. However, despite highly similar structures and the recognition of highly similar DNA sequences, members of a TF family can exhibit diverse biological functions. In order to shed light on the determinants of TF function, we focused on the FOXO family of TFs. FOXO proteins are involved in tumor suppression, due to their function in cell cycle arrest, apoptosis, response to ROS, senescence, but are also engaged in cellular differentiation (Katayama et al., 2008; Medema et al., 2000; Tran et al., 2002; Brunet et al., 1999; Essafi et al., 2005; de Keizer et al., 2010; Jiramongkol and Lam, 2020; Essers et al., 2005). Many of the FOXO functions are shared, but sill there are a few distinct and even opposite biological functions within FOXO proteins.

Recently, it was shown that while the sequence of the FH domain in the FOXO family is highly conserved, there are variations in their structures (Psenakova et al., 2019). In the present work, we aimed to compare the structures and dynamics of the FOXO FH domains and to ascertain whether any observed differences may affect FOXO key interactions. To this end, we studied the structures and dynamics of the FH domains all FOXO members, namely FOXO1, FOXO3, FOXO4, and FOXO6 in solution using NMR spectroscopy under identical experimental conditions and extensive MD simulations.

Overall, the structures and dynamics of the FH domains of all FOXO proteins are similar. Yet, we identified subtle differences in the structure and dynamics of the region connecting H2 and H3, which harbors a short helix H4 and a five amino acid insertion that is characteristic for FOXO proteins, despite the sequence being fully conserved. In the case of FOXO3, this region is dynamic on the ps-ns timescale, suggesting that H4 is not stably formed in FOXO3 in solution. Structural variations in this region were first proposed by Psenakova et al. (2019), based on a comparison of FOXO structures obtained by NMR spectroscopy and X-ray crystallography. Here we confirmed this notion for all FOXOs based on data obtained under identical experimental conditions. Functionally, H4 is not involved in DNA recognition in FOXO1 and FOXO3, but in FOXO4 Ser142 interacts with the phosphate group from the DNA backbone, thereby stabilizing the FOXO4-DNA complex (Boura et al., 2010). Thus, the differential dynamics of H4 of FOXO FH domains might be relevant for differential DNA binding and recruitment of FOXO proteins to different target genes. In a meta-analysis study aimed to identify direct FOXO targets through evolution *C. elegans* FOXO/DAF-16, *Drosophila* FOXO/DAF-16, mouse FOXO1 and FOXO3 and human FOXO3 were compared. This study found that FOXOs share targets across different tissues in mammals, and that function and identity of these shared mammalian targets are conserved in invertebrates. Isoform specific target genes have not been studied and discussed and remain elusive (Webb et al., 2016). At the structural level of FOXO proteins, additional contacts of residues from H4 with the DNA backbone might alter the dynamics of DNA recognition and binding. Furthermore, even differences in DNA binding affinity might allow different recruitment of co-regulators. All of this in turn might affect the transcriptional profile and be relevant for biological functions.

Using MD simulations, we were able to observe a conformational exchange process in FOXO3, in which Cys190 at the C-terminus of H2 loses contacts with Trp186 and instead contacts Trp157, localized in a flexible region that is N-terminal of H1. Intriguingly the former C-terminal residue on H2 is not conserved between FOXOs (Ser193 in FOXO1, Cys190 in FOXO3, Thr134 in FOXO4 and Tyr121 in FOXO6). This brought us to identify a common 3D-motif of sequence W-[2-5]-[S/T/C/Y] motif in all FOXO FH domains, where the former residue (Xaa) interacts via the side chain with the indole of a Trp residue. In the FH domains these interactions for Ser/Cys/Thr/Tyr with Trp are in the same range of energy but strongly vary in the relative orientation of the interacting side chains. In our quantum chemistry calculations (Fig. 5), the different conformations observed along our MD simulation studies of the motif W-[2-5]-[S/T/C/Y], yielded different energies for each of the FOXO FH domains when using the force-field based amino acid interaction (INTAA) web server (Barik, 2020). Thus, these four residues might have been found as equivalent solutions for the interaction with Trp along evolution and may constitute a way of introducing metastable interactions within intrinsically disorder proteins. The relevance of Trp in the interactome is clear: (i) it has the largest nonpolar (hydrophobic) area of all natural amino acids, (ii) its two-sided π electron density is polarizable and highly accessible for interaction with, for example, cations, and (iii) the indole N–H moiety in Trp is a strong hydrogen-bond donor (Barik, 2020). Although these motifs might not be related to the binding to DNA, such non-covalent hydrophobic interactions are crucial for protein stability, function and ligand binding. Indeed, they are the more abundant non-covalent interactions in the interaction of small molecules with proteins (Ferreira de Freitas and Schapira, 2017). Noteworthy, little is known to this point about the interaction between sulfur-containing (i.e. Cys) and aromatic amino acids (i.e. Trp), with only few available theoretical studies using simplified models (Ringer et al., 2007; Gomez-Tamayo et al., 2016). Thus, our study represents one of the few works so far reporting some geometrical features for the interaction between Ser/Cys/Thr and Trp side chains in the context of protein dynamics and conformational exchange processes.

One way that FOXO4 is regulated is by the intramolecular interaction of the CR3 with its corresponding FH domain (Bourgeois et al., 2021; Kim et al., 2021). This was shown to cause an auto-inhibited state, which impairs DNA binding. Binding of the CR3 to transcriptional co-activators can release this auto-inhibition and induces FOXO4 mediated transcription (Bourgeois et al., 2021). Wang et al. reported an intramolecular interaction of the FOXO3 CR3 with its respective FH domain, the role of this interaction in regulation of p53 binding, and hypothesized that these interactions could regulate intracellular localization of p53 (Wang et al., 2008). However, whether the FH-CR3 interaction is conserved among other FOXOs remained unclear. Here we show that the intramolecular interaction of FH and CR3 is conserved within the FOXO family, and that the regions within the FH domains, which are involved in CR3 binding are highly similar. Yet, whereas there are strong chemical shift perturbations of ^1^H,^15^N cross-peaks of FOXO1^FH^, FOXO4^FH^ or FOXO6^FH^, respectively upon addition of their corresponding CR3, there are only weak chemical shift perturbations found in ^1^H,^15^N cross-peaks of FOXO3^FH^, indicating a weaker interaction in FOXO3. Thus, it is likely that the auto-inhibitory mechanism is conserved among the FOXO family and might be less effective in FOXO3.

Lastly, we show that the FH – CR3 interaction can occur in an intermolecular manner involving different FOXOs. This might allow transcription regulation involving multiple FOXO proteins. We found that intermolecular interactions between FOXOs vary in strength, with the CR3 of FOXO4 showing the strongest binding to other FH domains, and FOXO3 showing the weakest interactions. For all FOXOs, residues in the N-terminal loop and H3 of the FH domain of FOXO proteins are affected most upon CR3 binding. This is intriguing since the N-terminal Trp residue shows different conformations in the MD simulations of the FOXO FH domains, which might account for the differential CR3 binding.

The biological function of FOXO transcription factors is regulated by the binding of transcriptional co-activators such as β-catenin (Essers et al., 2005; Hoogeboom et al., 2008; Li et al., 2021b; Liu et al., 2015). β-catenin is involved in cell adhesion, proliferation and development (Clevers and Nusse, 2012; MacDonald et al., 2009; Valenta et al., 2012). At least three FOXO family members were shown to interact with β-catenin and recently it was shown that this interaction is mediated via multivalent interactions of the FH domain, the CR2 and CR3 of FOXO4 with β-catenin (Bourgeois et al., 2021). Binding of β-catenin was shown to enhance FOXO4 transcriptional activity in response to oxidative stress (Essers et al., 2005). In the case of FOXO1, β-catenin was shown to be involved in oxidative stress-induced liver inflammation and necroptosis. Myeloid FOXO1 is phosphorylated by JNK in response to irradiation, subsequently translocates to the nucleus and cooperates with β-catenin to modulate the Hedgehog/Gli1/Snail pathway. Myeloid FOXO1 deficiency in turn leads to enhanced β-catenin activity and promoted Hedgehog/Gli1/Snail signaling, which reduced NEK7/NLRP3-driven liver inflammation and RIPK3-mediated necroptosis. (Li et al., 2021b) Binding of β-catenin to FOXO3 modulates epithelial-mesenchymal transition (EMT) by control of related genes in context of prostate cell cancer, which enables cells to migrate and invade (Liu et al., 2015). On molecular level it was shown that binding of the CR2 and CR3 of FOXO4 to β-catenin releases the auto-inhibiting interaction between the FH domain and the CR3 (Bourgeois et al., 2021). Our data on the intra-molecular interaction between the CR3 and FH domain of all FOXO proteins provides evidence that a similar mechanism can be envisaged for other FOXO proteins. The weaker intramolecular interaction between FH domain and CR3 of FOXO3 might make dissociation by β-catenin more likely.

FOXOs might form higher-order complexes, and these complexes might be important for transcription regulation. As an example, FOXO1 can dimerize upon binding to the palindromic DNA element DIV2 (Li et al., 2021a). The dimerization motif is located in W1, which was previously described to be involved in DNA binding directly via interaction with the backbone. In the structural models, the N-terminal Trp residue is located between the DNA and H4, without directly making contacts. Not only FOXO1, but also FOXO3 can bind to the DIV2 sites as dimer and also the other FOX protein FOXM1, FOXI1 can bind DIV2 sites as dimer. Interestingly, FOXC2 binds the DIV2 binding site as a monomer. The binding of other FOXO proteins was not covered in this study (Li et al., 2021a). At least in FOXO6 the amino acids responsible for dimerization are conserved, whereas the Gln in wing1 is substituted to His in FOXO4. The interaction between the FOXO FH and CR3 domains might contribute to the regulation of dimerization as well as the formation of hetero-oligomeric complexes comprising different FOXO isoforms. Heterodimerization of TFs is a common mechanism to regulate DNA binding specificity and enables alternative modes of DNA recognition (Vranken et al., 2005). Heterodimerization of FOXO proteins might enhance add another layer of regulation and tighten the control of FOXO transcription on one hand and might enable alternative DNA recognition on the other hand. Our findings of FH-CR3 interactions provide the basis for further studies to elucidate their effect on FOXO DNA recognition and binding in cells.

In summary, we found distinct variations in the structure and dynamics of the FH domain of FOXO proteins. Especially the FH domain of FOXO3 exhibits relatively higher flexibility and more pronounced ps-ns dynamics, which may be due to Cys190 at the C-terminus of H2 and the conformational exchange between binding to the Trp187 and binding to Trp157 located N-terminal of H1. We describe intra- and intermolecular interactions between the CR3s and FH domains of FOXO proteins of different affinities. We present the first comparison study of the structure and dynamics of all FOXO proteins and their intramolecular interactions. Additionally, we show that FOXO proteins can interact with each other via FH – CR3 interactions at different strengths. Taken together, this study provides essential clues for better understanding of the functional difference of FOXO proteins in biomolecular pathways.

## 4 Material and methods

### 4.1 Plasmids

Expression constructs for the fragments of human FOXO1 (Uniprot ID Q12778) spanned amino acids143 to 270 (FOXO1^FH^) and amino acids 587 to 636 (FOXO1^CR3^), for human FOXO3 (Uniprot ID O43524) from amino acid 141 to 260 (FOXO3^FH^) and from amino acid 593 to 646 (FOXO3^CR3^) and for human FOXO6 (Uniprot ID A8MYZ6) from amino acid 71 to 191 (FOXO6^FH^) and from amino acid 418 to 472 (FOXO6^CR3^). These constructs were generated by synthesis of the *Escherichia.coli* codon optimized DNA (Genscript) that were sub-cloned into pETM11-ZZ-His6 vector via *Nco*I/*Bam*HI restriction. Expression constructs for FOXO4 (Uniprot ID P98177) were described previously (Pon and Marra, 2015).

### 4.2 Protein expression and purification

For expression of recombinant unlabeled or ^15^N labeled or ^15^N–^13^C labeled His_6_-protein A-tagged proteins, the bacterial expression vectors were transformed into *E*. *coli* BL21-DE3 Star strain competent cells and grown in M9 minimal medium supplemented with either 6 ​g of unlabeled glucose (Roth) or 2 ​g of uniformly ^13^C-labeled glucose (Cambridge Isotope Laboratories) and 1 ​g of ^15^NH_4_Cl (Sigma). Each of the 1 ​L expression cultures were grown until an OD (600 ​nm) of 0.7 was measured, after which protein expression was induced by addition of 0.5 ​mM IPTG and allowed to continue for 16 ​h at 20 ​°C for FH constructs or 15 ​°C for CR3 constructs. After pelleting the cells via centrifugation at *ca.* 8000×*g*, the pellet was resuspended and lysed by sonication in either a denaturing lysis buffer (50 ​mM Tris-HCl pH 7.5, 150 ​mM NaCl, 20 ​mM Imidazole, 6 ​M urea) for CR3 constructs or in a non-denaturing lysis buffer (50 ​mM Tris-HCl pH 7.5, 150 ​mM NaCl, 20 ​mM Imidazole, 2 ​mM tris(2-carboxyethyl)phosphine) for FH constructs. His_6_-protein A-tagged recombinant proteins were then purified using Ni-NTA agarose (Qiagen) and the His_6_-protein A-tag was cleaved with His_6_-tagged TEV protease treatment. Untagged proteins were then isolated performing a second affinity purification using Ni-NTA beads. A final size exclusion chromatography purification step was performed in the buffer of interest on a gel filtration column (Superdex 75 increase, GE Healthcare). Protein concentrations were estimated based on their absorbance at 280 ​nm.

### 4.3 NMR measurements

NMR experiments were performed at 25 ​°C on 700 ​MHz spectrometer equipped with a TCI triple-resonance cryo-probe or on a Bruker 600 ​MHz Avance Neo NMR spectrometer equipped with a TXI room temperature probe.

Alongside the 2D ^1^H, ^15^N HSQC spectrum, the following three-dimensional spectra were acquired for NMR resonance assignment of FOXO6^FH^: HNCACB, CBCA(CO)NH, (H)CC(CO)NH-TOCSY and HCCH-TOCSY using 550 ​μM ^13^C^15^N labeled FOXO6^FH^. Previous assignments for FOXO1^FH^ (BMRB 27894) and FOXO3^FH^ (BMRB 15939) were confirmed using HNCACB and CBCA(CO)NH spectra using 500 ​μM ^13^C^15^N labeled FOXO1^FH^ or using 300 ​μM ^13^C^15^N labeled FOXO3^FH^ respectively. Resonance assignments have been deposited under BMRB accession number XXX (will be updated upon acceptance).

For resonance assignment of FOXO1^CR3^, FOXO3^CR3^ and FOXO6^CR3^, HN(CA)NNH, H(NCA)NNH, HNCACB and CBCA(CO)NH were recorded using 300–400 ​μM ^13^C^15^N labeled FOXO1^CR3^, FOXO3^CR3^ and FOXO6^CR3^ respectively. Resonance assignments have been deposited under BMRB accession number 51426. Spectra were processed using TOPSPIN 4.1 (Bruker) and analyzed using CcpNmr 2.5.

### 4.4 Data analysis

The CSPs was calculated according to the following equation (1):

$$CSP=\sqrt{{\left(\delta H\right)}^{2}+\frac{{\left(\Delta \delta N\right)}^{2}}{7}}$$

where ΔδH and ΔδN indicate the chemical shift changes of the amide proton and nitrogen, respectively, which were measured using CcpNMR 2.5 (Vranken et al., 2005).

The correlation matrix of FOXO FH domains was calculated using the Pearson function following equation (2):

$$r=\frac{\sum \left(x-\bar{x}\right)\left(y-\bar{y}\right)}{\sqrt{\sum {\left(x-\bar{x}\right)}^{2}\sum {\left(y-\bar{y}\right)}^{2}}}$$

where x and y are the sample means of chemical shifts of the respective chemical shifts.

RMSD values for the FOXO structures were obtained used the PyMol align method using only backbone atoms and zero cycles of outlier detection. Pairwise alignment and RMSD calculations were performed using the ColorByRMSD python module for PyMol using the align method and backbone atoms.

### 4.5 ^15^N spin relaxation measurements and error analysis

NMR relaxation experiments for all FOXO FH domains were recorded using ^15^N labeled samples at 300 ​μM protein concentration in a buffer containing 50 ​mM NaH_2_PO_4_/Na_2_HPO_4_ (pH 6.5), 0.04% NaN_3,_ 2 ​mM DTT and 10 %D_2_O. ^15^N R_1_ values were measured using the HSQCT1ETF3GPSI3D.2 Bruker pulse sequence (8 scans, 256 ​× ​1024 complex points in and spectral widths of 9615.410 ​× ​1825.051 ​Hz in *t*_1_ x *t*_2_, respectively, using delays of 0.01, 2.00, 0.0308, 1.273, 0.063, 0.806, 0.114, 0.507, 0.193, and 0.315 ​s, with the 0.507-s delay time duplicated for error analysis. ^15^N R_1ρ_ values were measured using the HSQCTRETF3GPSI3D Bruker pulse sequence (16 scans with the same spectral parameters as above), using delays 0.01, 0.2, 0.012, 0.131, 0.0151, 0.086, 0.0199, 0.0575, 0.0274, 0.0575 and 0.0392 ​s ^15^N{^1^H} heteronuclear NOE was measured using the HSQCNOEF3GPSI Bruker pulse sequence (32 scans, 256 points in F1, 1024 points in F2, 1278.772 ​Hz spectral width in F1, 9615.385 ​Hz spectral width in F2, 1024 transients) using 3 ​s saturation period/interscan delay. For the NMR binding assays, all protein samples were equilibrated in the same buffer containing 50 ​mM NaH_2_PO_4_/Na_2_HPO_4_ (pH 6.5), 0.04% NaN_3_ and 5 ​mM DTT. ^1^H, ^15^N HSQC experiments were recorded according to the NMR section.

The spin relaxation datasets were each recorded in an interleaved manner as pseudo-3D experiments in which the variable relaxation delays (*T*_1_, *T*_1ρ_) or ^1^H pulse saturation (hetNOE) were encoded in the pseudo-third dimension. In the *T*_1_ and *T*_1ρ_ experiments, 11 different 2D planes were recorded with maximum relaxation delays of 2 ​s and 0.2 ​s, respectively, and two duplicate measurements were collected to compute the error in measured intensities. The data were processed in NMRPipe (Delaglio et al., 1995), analyzed with NMRFAM-Sparky (Lee et al., 2015), and lineshapes were fit with FuDA (Vallurupalli et al., 2008) to extract the intensities.

*T*_1_ and *T*_1ρ_ data were fit to a two-parameter exponential decay, *I*_0_e^−*R*t^, where *I*_0_ is the fitted intensity at zero time and *R* is fitted rate of decay, with the reported *R*_1_ and *R*_1ρ_ rates referring to 1/*T*_1_ and 1/*T*_1ρ_, respectively. A^15^N spin-lock field strength of 1.9 ​kHz was employed in the *T*_1ρ_ experiment, and the *R*_2_ rates were corrected for the off-resonance tilted field based on equation (3):

$$R_{2}=\frac{R_{1\rho }}{sin\theta^{2}}-\frac{R_{1}}{tan\theta^{2}}$$

where θ ​= ​arctan(ω_1_/Ω), with ω_1_ equal to the spin-lock field strength (in rad/s) and Ω the ^15^N resonance offset. The fitted *R*_1_ and *R*_1ρ_ rates were obtained via least-squares minimization using the standard Levenberg-Marquardt algorithm included in the lmfit Python package (https://lmfit.github.io/lmfit-py/). The errors in measured peak intensities were derived from the pooled standard deviation of the duplicated delay measurements, with the noise computed as: $\sqrt{\underset{i}{\sum }{\left(s_{i,1}-s_{i,2}\right)}^{2}/2k}$ where *i* refers to the peak associated with residue *i, s*_*i*,1_ and s_i,2_ refer to the signal intensity associated with the two duplicated time delays for residue *i*, and *k* refers to the total number of peaks in the analysis. Errors in the fitted relaxation rates were obtained via Monte Carlo analyses performed with an in-house Python script. In this analysis, 1000 synthetic datasets were generated for each residue in which each intensity value was randomly sampled from a normal distribution centered at the measured intensity with a width corresponding to two times the noise (as calculated above). The least-squares minimization procedure was performed on each of these 1000 synthetic datasets, and the error in the fitted rate constant was calculated by fitting the histogram of the best-fit values to a normal distribution: $\left(A/\sigma \sqrt{2\pi }\right)exp$, where μ is the center of the distribution, σ the width of the distribution, and *A* the amplitude for normalization. The error was obtained from σ, which corresponds to the standard deviation of a normally distributed parameter and the reported errors in Fig. 3 are 2σ or the 95% confidence interval. For the hetNOE dataset, the error was calculated based on the intensity of the peak in the reference plane and the error in intensity derived from fitting the lineshape with FuDA.

### 4.6 AlphaFold2

AlphaFold2 prediction were performed using the easy to use AlphaFold2 (Jumper et al., 2021) a protein structure prediction pipeline, with an API hosted at the Söding lab based on the MMseq2 server (Bourgeois et al., 2021) for the multiple sequence alignment creation. The sequence was chosen according the fragment size described in the Plasmids section.

### 4.7 Molecular dynamics simulations

The NMR-solved structure of the human FH domain of the isoforms of 1, 3 and 4 of FOXO were obtained from the Protein Data Bank (PDB ids. 6QVW, 2K86 and 1E17, respectively) and the theoretical model (version 1) for the FH domain of human FOXO6 was gotten from the AlphaFold2 Protein Structure Database (Jumper et al., 2021). The solution structure of the FOXO4 FH-CR3 complex has been solved as described previously (Bourgeois et al., 2021). In the simulations, all proteins were described by the AMBER ff19SB force field (Kim et al., 2021)) and the solvent molecules were represented as OPC water molecules (Furuyama et al., 2000).Periodic boundary conditions were employed with a cutoff of 10 ​Å for non-bonded interactions and the Particle Mesh Ewald method for long-range electrostatics was used. All the MD simulations were run using the Amber20 software package (Case HMA et al., 2021).

The protonation state of each of the residues of the proteins were fixed at pH 6.5 by means of the server H++, version 3.2 (Biggs et al., 1999, 2001; Kops et al., 1999). The proteins were embedded in a box of OPC water molecules (minimum distance of 12 ​Å from the solute to the boundaries of the box) and the net charge of the system was set to zero by neutralization with Cl-ions using the program tLEaP included in AmberTools21 (Case HMA et al., 2021). Then, the system was minimized in three stages: protons, then solvent molecules and counter ions, and finally, the whole system. Harmonic restraints of 200 ​kcal ​mol^−1^ Å^−2^ were imposed to the rest of the atoms in each stage and the system was thermally equilibrated for 20 ps from 100 ​K to 298 ​K using the Langevin thermostat. The harmonic restraint was removed in 5 steps where the system equilibrated from a NVT to an NPT ensemble for 100 ps. Then, each of the systems was further simulated for 500 ns in three independent simulations (3 ​× ​500 ns, 1.5 μs total time per system) using the Langevin thermostat at 298 ​K and with a time step of 0.2 fs. MD simulation trajectory analysis was performed using the program cpptraj.

The interaction energy between the terminal residues of the Xaa-(Yaa)_3-5_-W motif in the different isoforms was competed following the cluster method and using the counterpoise approach (Boys and Bernardi, 1970; Simon et al., 1996): first, from a snapshot of the MD simulations, the Cartesian coordinates of the two residues were taken and the N- and C-termini were capped with neutral functional groups (Ac and N-methyl ester). Second, the hydrogen atoms of the resulting system were optimized in gas phase using BP86-D3/def2SVP with the quantum chemistry program Orca (Neese et al., 2020) whereas the rest of the atoms (heavy atoms) were constrained. And third, the interaction energy was computed at the DFT level of theory using the Austin-Frisch-Petersson functional with dispersion (AFPD) (Weigelt et al., 2000) and a large basis set (6-311+G(2d,p)). The latter quantum mechanics calculations were run using Gaussian16, v.01. Statistical analysis of interaction energies was performed using GraphPad Prism 8 via Kruskal-Wallis test and Dunn's multiple comparisons test.

## Accession numbers

Protein Data Bank accession nos. **6QVW**, **2K86** and **1E17.** Uniprot accession nos. Q12778, O43524, P98177 and **A8MYZ6.**

## CRediT authorship contribution statement

**Emil Spreitzer:** Conceptualization, Methodology, Formal analysis, Investigation, Writing – original draft, Writing – review & editing, Visualization. **T. Reid Alderson:** Formal analysis, Conceptualization, Writing – review & editing, Supervision. **Benjamin Bourgeois:** Conceptualization, Writing – review & editing, Supervision. **Loretta Eggenreich:** Conceptualization, Investigation, Writing – review & editing, Visualization. **Hermann Habacher:** Investigation, Writing – review & editing, Visualization. **Greta Brahmersdorfer:** Conceptualization, Investigation, Writing – review & editing. **Iva Pritišanac:** Writing – review & editing, Visualization. **Pedro A. Sánchez-Murcia:** Conceptualization, Methodology, Formal analysis, Investigation, Writing – review & editing, Visualization, Resources, Data curation. **Tobias Madl:** Conceptualization, Methodology, Writing – review & editing, Resources, Supervision, Funding acquisition.

## Declaration of competing interest

The authors declare that they have no known competing financial interests or personal relationships that could have appeared to influence the work reported in this paper.
