# Supplementary material for: FOXO transcription factors differ in their dynamics and intra/intermolecular interactions
Source: Curr Res Struct Biol. 2022 Apr 27;4:118–33. doi: 10.1016/j.crstbi.2022.04.001 (PMC9097636; doi:10.1016/j.crstbi.2022.04.001)
Supplement: Multimedia component 1 [file mmc1.docx]

**FOXO transcription factors differ in their dynamics and**

**intra/intermolecular interactions**

**Supplementary Data**

Emil Spreitzer^1^, T. Reid Alderson^1^, Benjamin Bourgeois^1^, Loretta Eggenreich^1^, Hermann Habacher^1^, Greta Brahmersdorfer^1^, Iva Pritišanac^1^, Pedro A. Sánchez-Murcia^2^, Tobias Madl^1,3^

^1^ Gottfried Schatz Research Center for Cell Signaling, Metabolism and Aging, Molecular Biology and Biochemistry, Medical University of Graz, Graz, Austria

^2^ Division of Physiological Chemistry, Otto-Loewi Research Center, Medical University of Graz, Graz, Austria

^3^ BioTechMed-Graz, Graz, Austria

Corresponding Author: tobias.madl@medunigraz.at, +43/316/385-71972


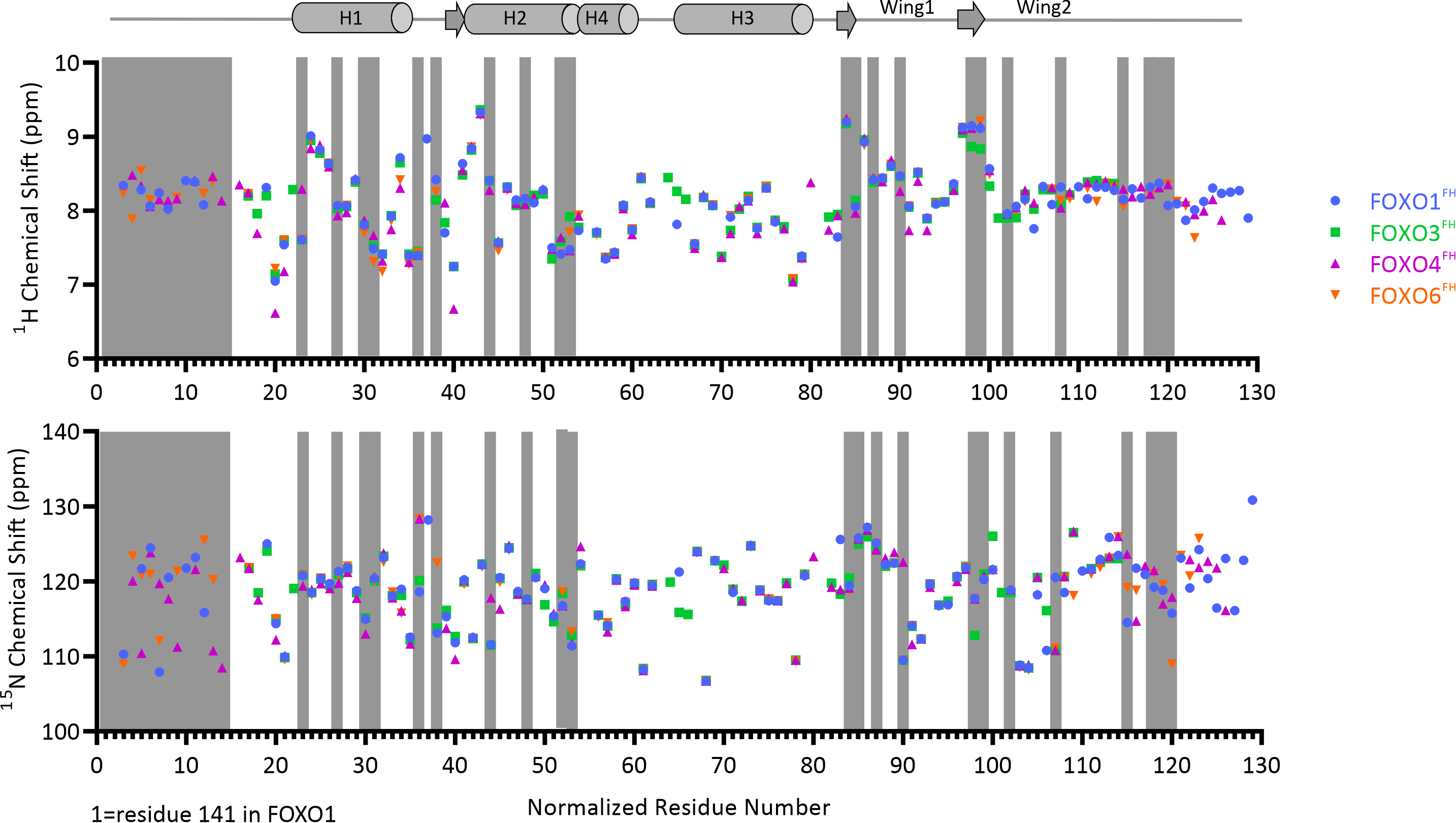


**Figure S1: ^1^H and ^15^N chemical shifts of FOXO FH domains.** ^1^H chemical shift in the upper panel or ^15^N chemical shift in the lower panel of the ^1^H,^15^N HSQC cross-peaks of FOXO FH domains. The residues numbers are normalized to 1 from residue 141 corresponding to FOXO1 numbering (residue 138 in FOXO3, residue 84 in FOXO4, residue 69 in FOXO6). Grey bars represent non-conserved residues.


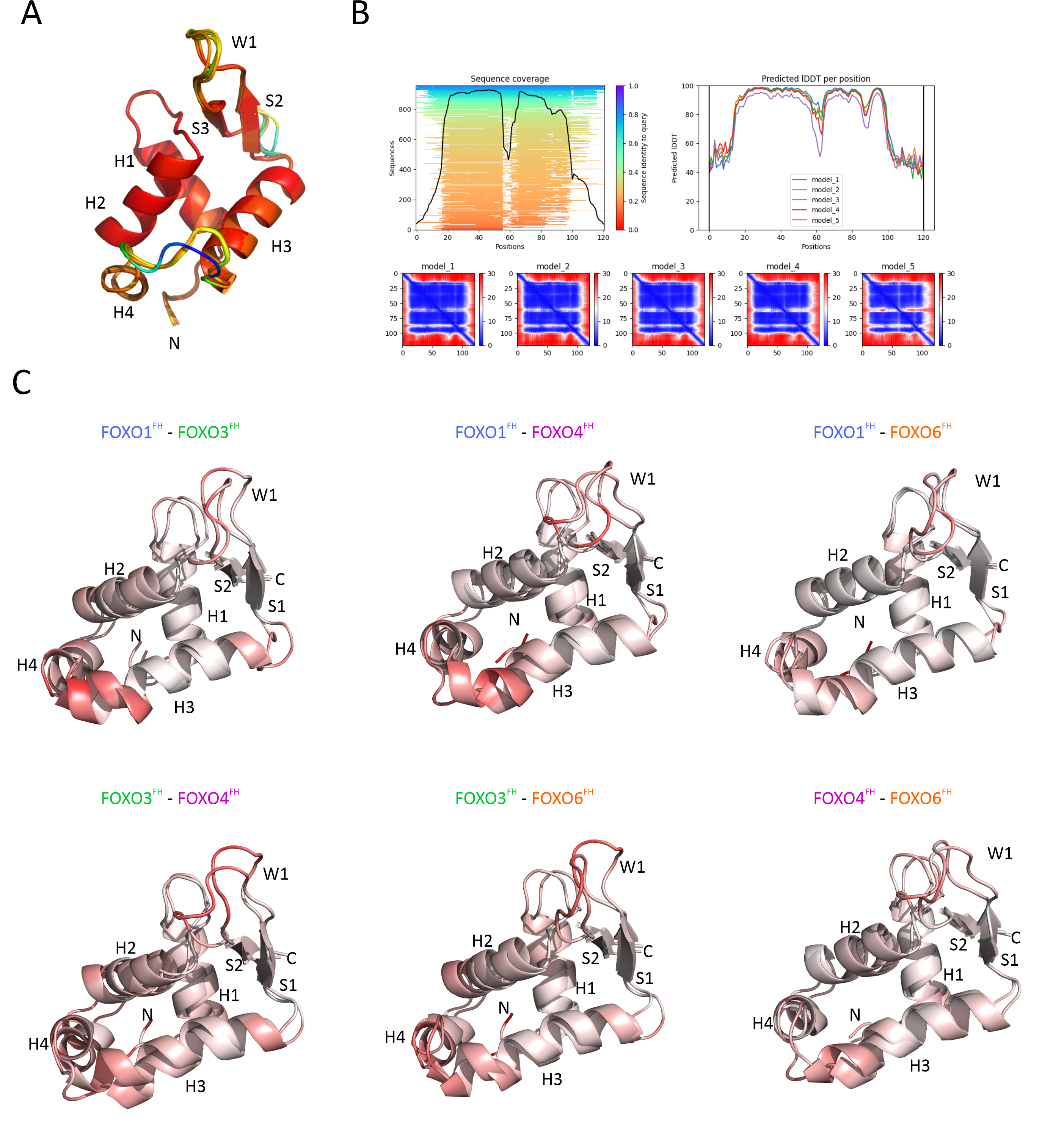


**Figure S2: Pairwise superimposition of FOXO FH domains:** (A) Superimposition of five AlphaFold2 predictions of FOXO6^FH^ based on amino acid sequence (71-191). (B) Sequence coverage, predicted local superposition-free score (pIDDT) and contact map of the AlphaFold2 prediction for FOXO6^FH^. (C) Comparison of FOXO FH domain structures: FOXO1 vs. FOXO3, FOXO1 vs. FOXO4, FOXO1 vs. FOXO6, FOXO3 vs. FOXO4, FOXO3 vs. FOXO6 and FOXO4 vs. FOXO6 colored by RMSD from 0 Å (white) to 10 Å (red).


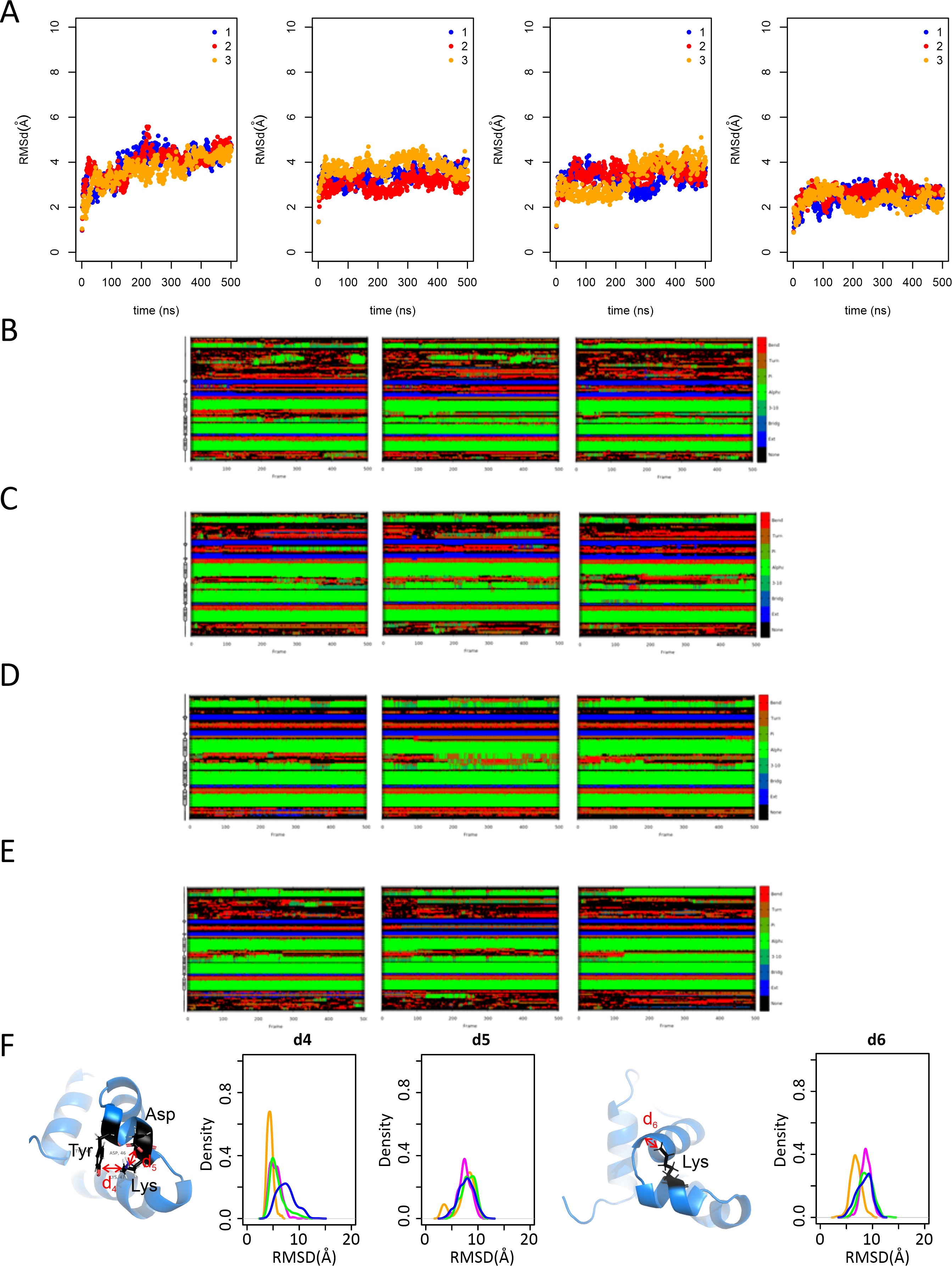


**Figure S4 RMSD and Secondary Structure Elements Derived from Molecular Dynamics Simulations.** A) RMSD analysis of three independent 500 ns simulations for FOXO1^FH (159-244)^, FOXO3^FH (156-241)^, FOXO4^FH (100-185)^ and FOXO6^FH (97-182)^ respectively. Secondary structure analysis for B) FOXO1^FH^, C) FOXO3^FH^, D) FOXO4^FH^ and E) FOXO6^FH^. The frames from the MD simulation are shown along the x-axis and residues are shown along the y-axis. Secondary structure types are color coded. F) Distance distribution within H4 (d_4_, d_5_) and between H2 and H4 of FOXO1 (blue), FOXO3 (green), FOXO4 (magenta) and FOXO6 (orange). Note that d_4_ and d_5_ correspond to different distance measurements in Figure 4.


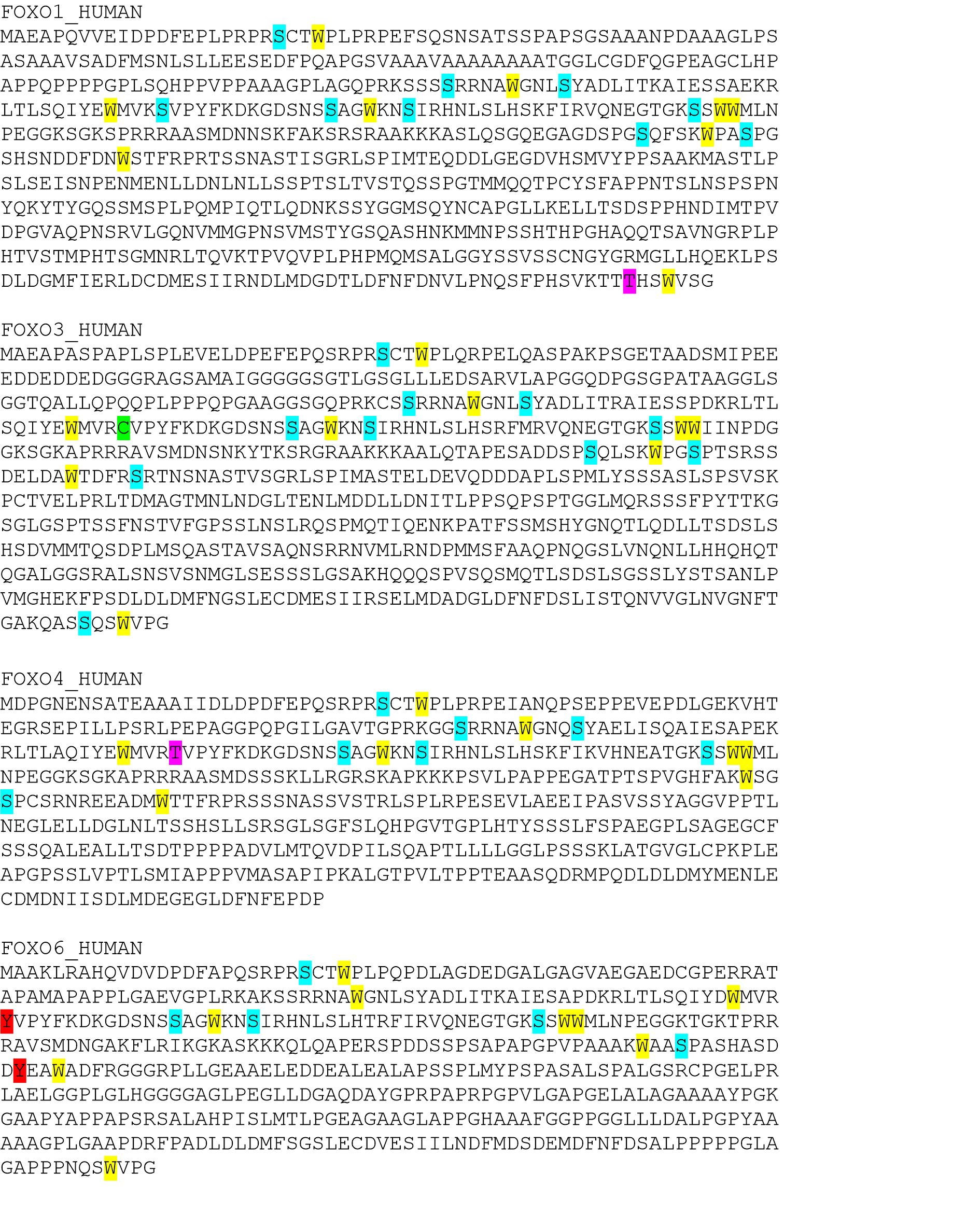


**Figure S5. FOXO Sequences highlighting W-.[2-5]-[S/T/C/Y] motifs throughout sequence.** Sequences of FOXO1, FOXO3, FOXO4 and FOXO6 respectively with Ser, Cys, Thr and Tyr located in W-.[2-5]-[S/T/C/Y] motif highlighted in blue, green, magenta, red respectively and all Trp residues highlighted in yellow.


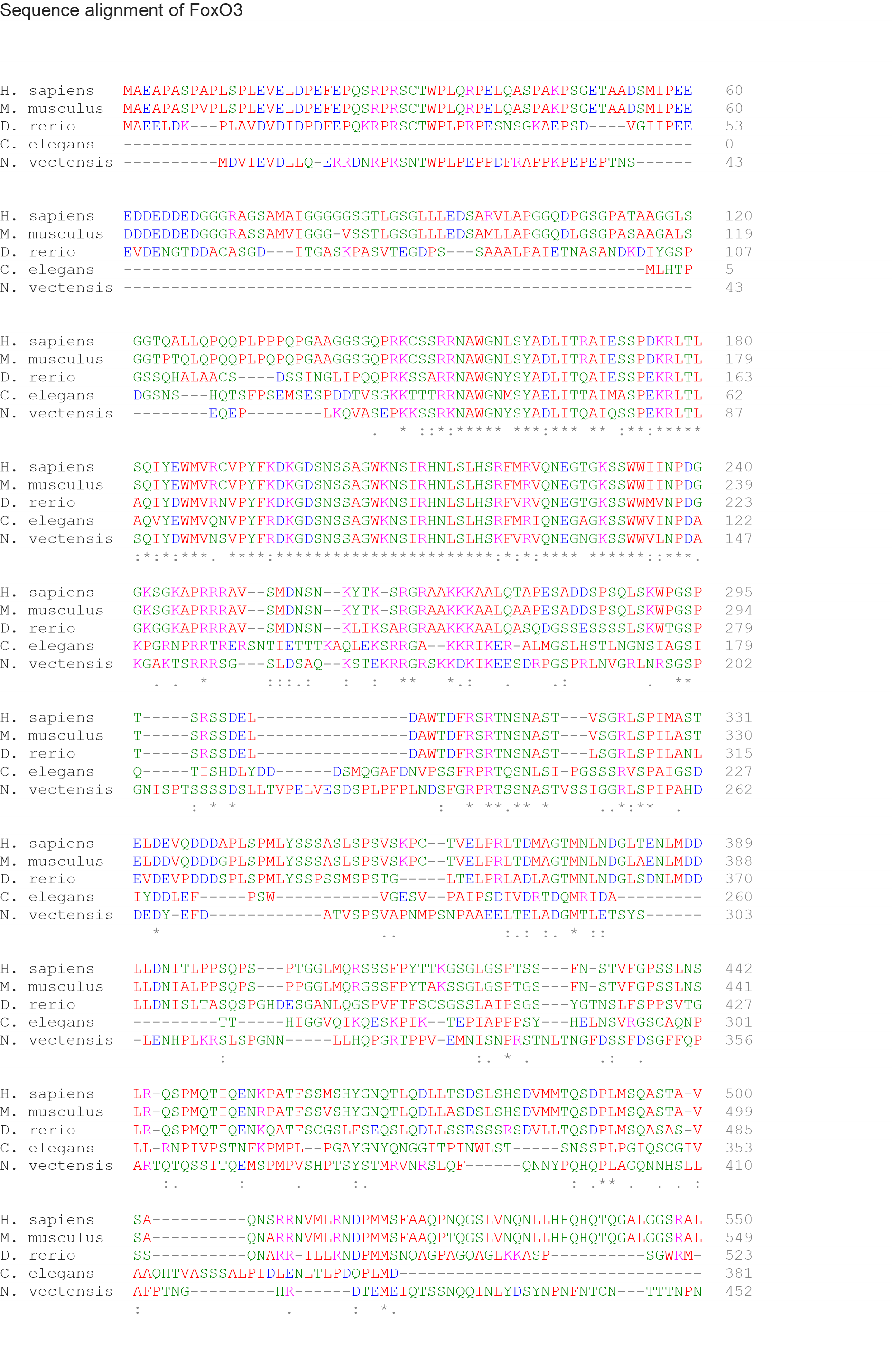


**Figure S6. FOXO3 Sequence Alignment highlighting W-.[2-5]-[S/T/C/Y] motifs throughout evolution**.


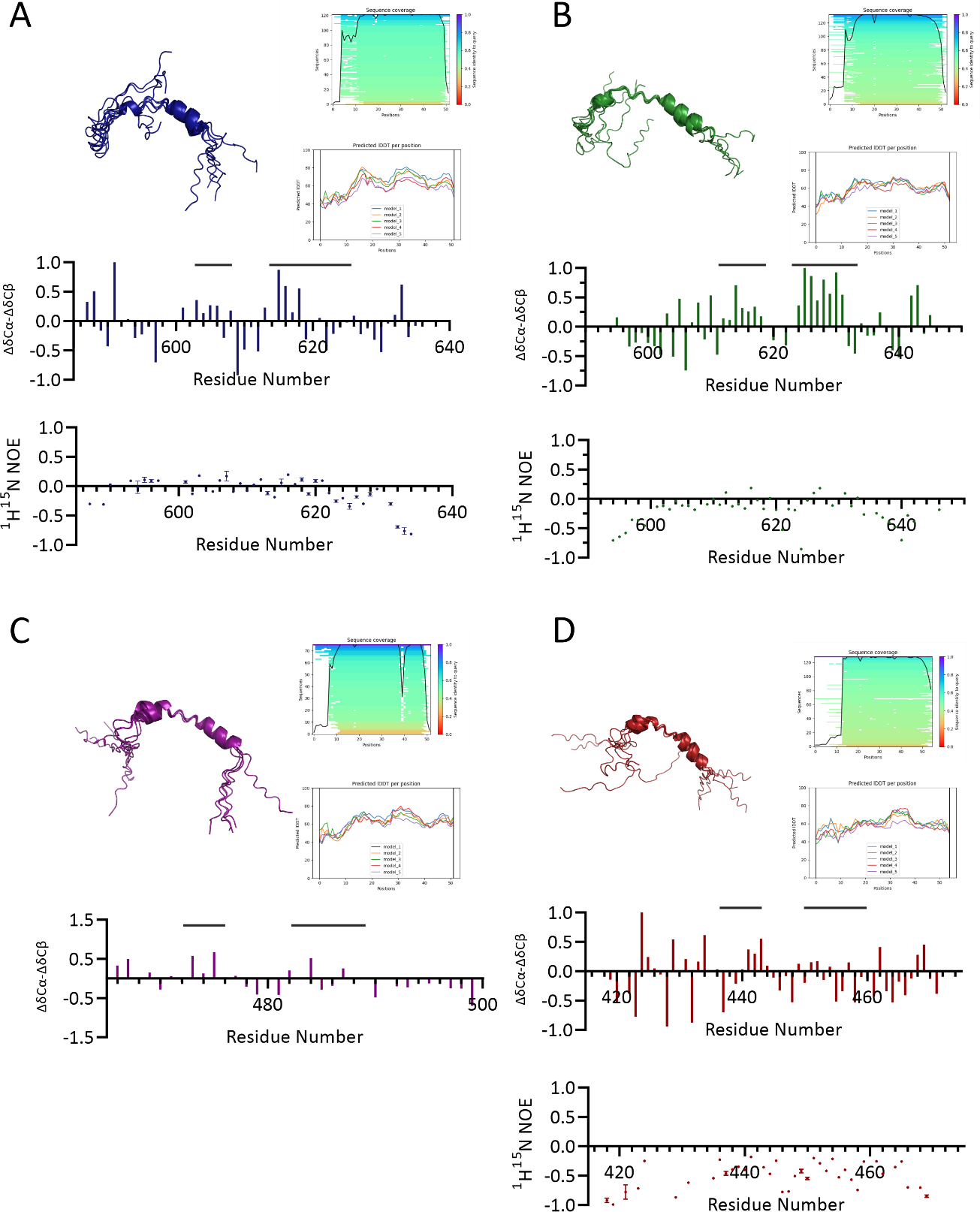


**Figure S7: AlphaFold2 structural predictions of FOXO CR3s and secondary structure propensity measured by NMR.** Five AlphaFold2 Prediction models are shown in superimposition. The corresponding sequence coverage and predicted IDDT are shown next to the structures. Below the AlphaFold2 structures, experimentally measured secondary chemical shifts and ^15^N{^1^H} heteronuclear NOE of the isolated A) FOXO1^CR3^, B) FOXO3^CR3^, C) FOXO4^CR3^ and D) FOXO6^CR3^ regions are shown.


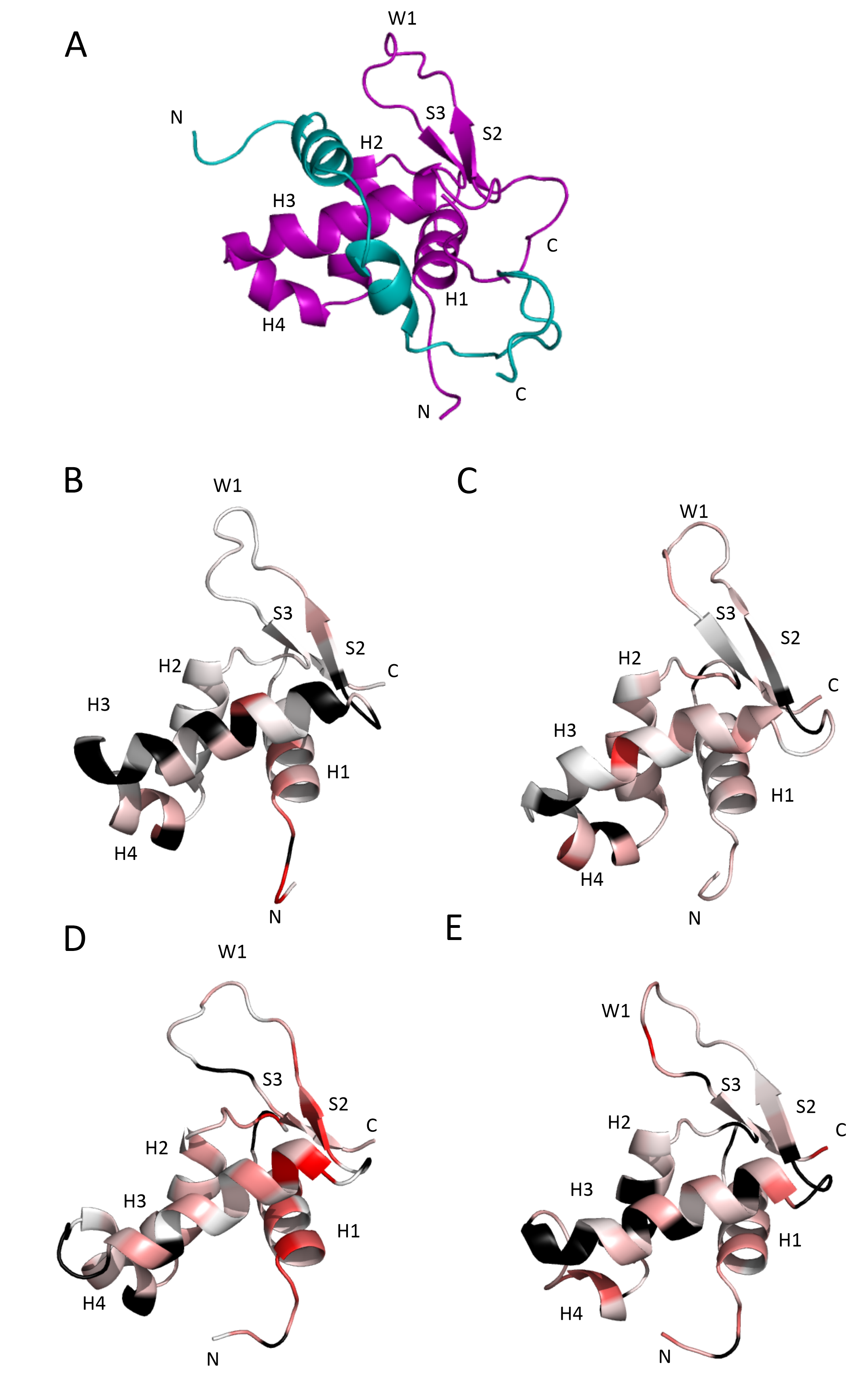


**Figure S8: FOXO FH domain structural models colored by CSPs induced by CR3 binding.** A) Solution structure of FOXO4^FH^ in complex with FOXO4^CR3^ with the CR3 shown in teal and FH domain in purple. Structural models of B) FOXO1^FH^, C) FOXO3^FH^, D) FOXO4^FH^ and E) FOXO6^FH^ colored based on CSPs upon binding to their respective CR3. Colored from 0 (white) to 0.1 (red) ^1^H^15^N normalized ppm. Unassigned residues are colored black.


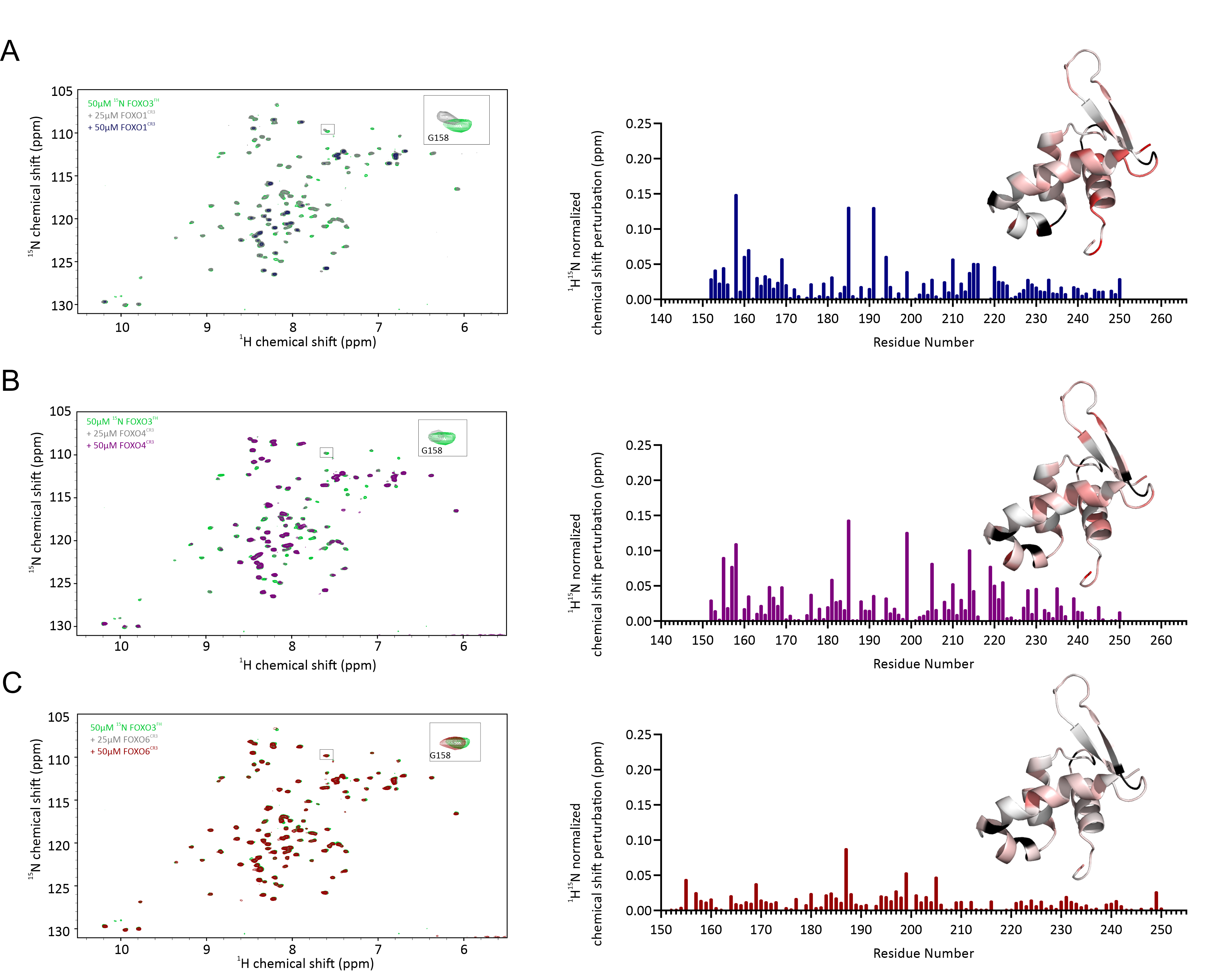


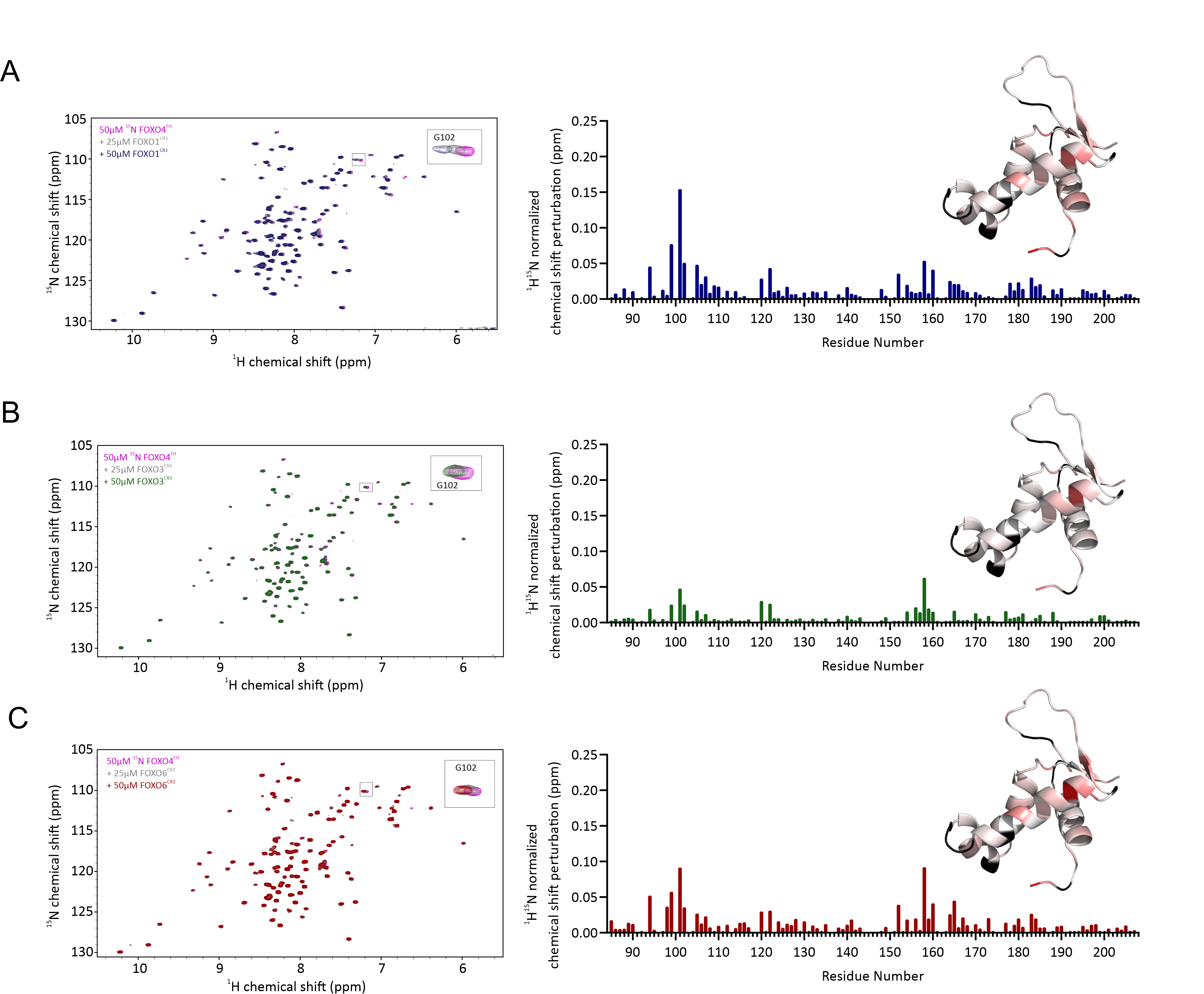


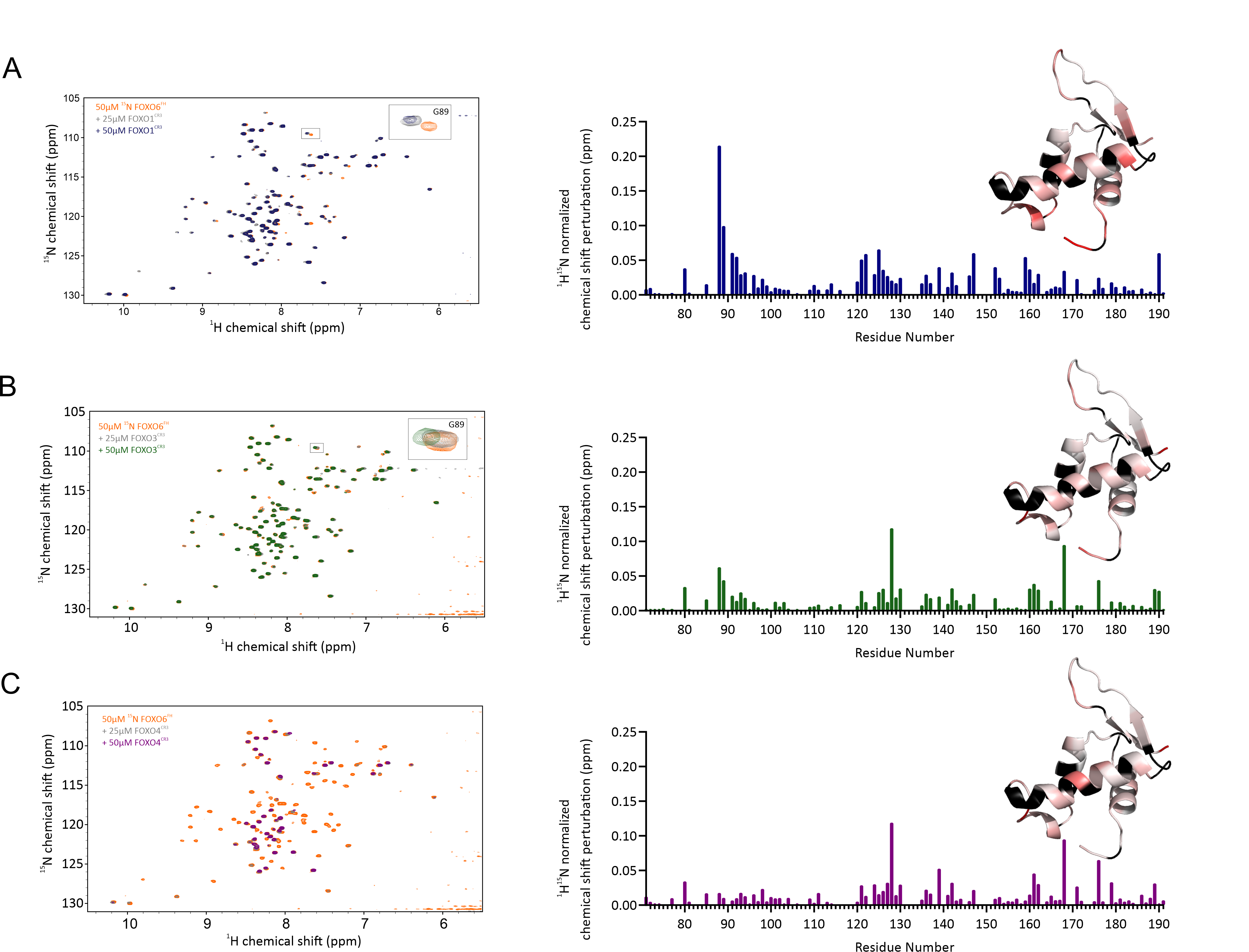


**Figure S9-12: NMR binding studies of intramolecular FH – CR3 interactions.**

**Figure S9.** (A–C) 2D ^1^H,^15^N HSQC spectra of 50µM ^15^N labelled FOXO1^FH^ (blue) in the presence of 25 µM (grey) and 50 µM FOXO3^CR3^ (dark green), FOXO4^CR3^ (dark blue), FOXO6^CR3^ (dark red), respectively and corresponding chemical shift perturbation quantification analysis.

**Figure S10.** A–C) 2D ^1^H,^15^N HSQC spectra of 50µM ^15^N labelled FOXO3^FH^ (green) in the presence of 25 µM (grey) and 50 µM FOXO1^CR3^ (dark blue), FOXO4^CR3^ (dark violet), FOXO6^CR3^ (dark red), respectively and corresponding Chemical shift perturbation quantification analysis.

**Figure S11.** (A–C) 2D ^1^H,^15^N HSQC spectra of 50µM ^15^N labelled FOXO4^FH^ (magenta) in the presence of 25 µM (grey) and 50 µM FOXO1^CR3^ (dark blue), FOXO3^CR3^ (dark green), FOXO6^CR3^ (dark red), respectively and corresponding Chemical shift perturbation quantification analysis.

**Figure S12.** (A–C) 2D ^1^H^15^N HSQC spectra of 50µM ^15^N labelled FOXO6^FH^ (magenta) in the presence of 25 µM (grey) and 50 µM FOXO1^CR3^ (dark blue), FOXO3^CR3^ (dark green), FOXO4^CR3^ (dark violet), respectively. The corresponding chemical shift perturbation quantification is shown next to the spectra.
